# Supplementary material for: Evaluation of an integrated intervention to reduce psychological distress and intimate partner violence in refugees: Results from the Nguvu cluster randomized feasibility trial
Source: PLoS One. 2021 Jun 18;16(6):e0252982. doi: 10.1371/journal.pone.0252982 (PMC8213126; doi:10.1371/journal.pone.0252982)
Supplement: S1 File — Model results describing participant characteristics that were associated with intervention completion and study attrition. (DOCX) [file pone.0252982.s001.docx]

**Supplementary File 1 (S1_File): Baseline correlates of intervention completion and study attrition**

|  | Intervention Completion (among Nguvu intervention participants)  OR (95% CI) | Study Attrition (in full sample)  OR (95% CI) |
| --- | --- | --- |
| Age (in years) | 1.02 (0.98-1.05) | 1.02 (0.99-1.06) |
| Education (in years) | 0.92 (0.83-1.02) | 1.06 (0.94-1.20) |
| Marital Status |  |  |
| Married and living with partner | REF | REF |
| Married, not living with partner | 1.00 (0.21-4.65) | 1.46 (0.40-5.37) |
| In relationship and living with partner | 0.75 (0.23-2.45) | 1.30 (0.36-4.72) |
| In relationship, not living with partner | 1.60 (0.61-4.21) | 0.82 (0.27-2.49) |
| Housing type |  |  |
| Grass and mud | REF | REF |
| Blocks and tend | 0.44 (0.03-6.70) | 0.93 (0.11-7.62) |
| Blocks and grass | 0.45 (0.05-4.45) | -- |
| Blocks and iron sheet | -- | -- |
| Any children |  |  |
| No | REF | REF |
| Yes | 1.05 (0.23-4.85) | 1.00 ( .- .) |
| Household size | 0.96 (0.87-1.06) | 0.99 (0.89-1.10) |
| Number of children | 1.12 (0.98-1.29) | 0.96 (0.83-1.11) |
| Did you go to school? |  |  |
| No | REF | REF |
| Yes | 0.79 (0.34-1.85) | 1.18 (0.49-2.83) |
| Literate |  |  |
| No | REF | REF |
| Yes | 1.16 (0.54-2.48) | 0.99 (0.44-2.22) |
| Religion |  |  |
| Catholic | REF | REF |
| Methodist and Free or United | 0.74 (0.23-2.36) | 1.34 (0.46-3.87) |
| Other Christian | 0.95 (0.41-2.19) | 0.80 (0.31-2.05) |
| Muslim | 1.11 (0.22-5.54) | 1.12 (0.21-6.05) |
| Other, please specify | 0.67 (0.14-3.19) | 0.97 (0.18-5.18) |
| Number of years living in Nyarugusu | 0.94 (0.88-1.01) | 0.99 (0.92-1.06) |
| Resettlement: listed on board |  |  |
| No | REF | REF |
| Yes | 0.79 (0.41-1.51) | 1.46 (0.72-2.95) |
| Resettlement: completed an interview |  |  |
| No | REF | REF |
| Yes | 0.67 (0.34-1.30) | 1.71 (0.85-3.46) |
| Resettlement: completed health screen |  |  |
| No | REF | REF |
| Yes | 0.71 (0.17-2.95) | 10.82 (3.65-32.06) |
| Resettlement: completed cultural orientation |  |  |
| No | REF | REF |
| Yes | 2.21 (0.22-21.71) | 21.64 (5.31-88.27) |
| Prefer to be resettled |  |  |
| No | REF | REF |
| Yes | 1.41 (0.19-10.25) | -- |
| Head of household would prefer to be resettled |  |  |
| No | REF | REF |
| Yes | 1.44 (0.28-7.56) | 0.55 (0.06-4.96) |
| Nguvu intervention group |  |  |
| 1A | REF | REF |
| 1B | 0.31 (0.06-1.61) | 0.89 (0.14-5.48) |
| 1C | 0.17 (0.02-1.23) | -- |
| 2A | 0.50 (0.09-2.81) | 0.22 (0.02-2.67) |
| 2B | 0.50 (0.10-2.60) | -- |
| 3A | 1.67 (0.29-9.71) | -- |
| 3B | 1.50 (0.25-8.84) | -- |
| 3C | 1.50 (0.25-8.84) | 0.18 (0.02-2.15) |
| 3D | 2.50 (0.21-29.25) | 0.40 (0.03-5.15) |
| 4A | 0.25 (0.05-1.36) | 0.40 (0.05-3.12) |
| 4B | 1.00 (0.18-5.46) | 0.18 (0.02-2.15) |
| 4C | 2.50 (0.21-29.25) | 0.40 (0.03-5.15) |
| 5A | 0.50 (0.10-2.60) | 0.18 (0.02-2.15) |
| 5B | 0.40 (0.07-2.37) | -- |
| 5C | 1.00 (0.16-6.25) | -- |
| Facilitator pair |  |  |
| A | REF | REF |
| B | 1.20 (0.41-3.54) | 0.35 (0.04-3.32) |
| C | 3.96 (1.48-10.60) | 0.35 (0.06-2.06) |
| D | 1.57 (0.58-4.25) | 1.12 (0.25-4.92) |
| E | 1.37 (0.51-3.70) | 1.12 (0.25-4.92) |
| PSTD symptoms | 0.99 (0.96-1.03) | 1.00 (0.96-1.04) |
| Depressive symptoms | 1.15 (0.52-2.53) | 1.17 (0.46-2.94) |
| Anxiety symptoms | 0.71 (0.37-1.36) | 0.90 (0.45-1.80) |
| Relationship quality | 0.84 (0.50-1.41) | 0.94 (0.53-1.67) |
| Frequency of psychological IPV | 0.83 (0.68-1.01) | 1.18 (0.97-1.44) |
| Frequency of physical IPV | 0.89 (0.67-1.20) | 1.16 (0.90-1.50) |
| Frequency of sexual IPV | 0.83 (0.71-0.96) | 1.05 (0.92-1.20) |
| Frequency of IPV | 0.74 (0.55-1.00) | 1.20 (0.93-1.55) |
| Functional impairment | 1.13 (0.72-1.78) | 1.05 (0.66-1.67) |
| Psychological distress | 0.94 (0.38-2.33) | 1.27 (0.46-3.47) |
