# Supplementary material for: Evaluation of an integrated intervention to reduce psychological distress and intimate partner violence in refugees: Results from the Nguvu cluster randomized feasibility trial
Source: PLoS One. 2021 Jun 18;16(6):e0252982. doi: 10.1371/journal.pone.0252982 (PMC8213126; doi:10.1371/journal.pone.0252982)
Supplement: S2 File — Study protocol approved by the Johns Hopkins Institutional Review Board. (DOCX) [file pone.0252982.s002.docx]

**JHSPH IRB Research Plan for New Data Collection**

**PI Name:** Wietse A. Tol, PhD

**Study Title:** Evaluating an Integrated Approach to Reduce Intimate Partner Violence and Improve Psychosocial Health in Refugees

**IRB No.:IRB00007219**

**PI Version No./Date:** 5/10-13-2017

**I. Aims of the Study:**

The overall aim of the study concerns the development and evaluation of an integrated approach that combines (a) mental health treatment with (b) an empowerment intervention, to reduce psychological distress and intimate partner violence against female refugees from the Democratic Republic of the Congo (DRC) in Tanzania.

Specifically, the study aims to:

1. Pilot measurement tools assessing identified priority mental health conditions, functional impairment and intimate partner violence in Nyarugusu refugee camp.
2. Develop and pilot implementation of the proposed integrated intervention combining and adapting elements of cognitive processing therapy (CPT) and empowerment counseling.
3. Conduct a randomized controlled trial of the intervention, to examine if the collaboratively developed intervention is associated with reductions in prioritized mental health problems and intimate partner violence, compared to a set of currently existing standard operating procedures
4. Conduct a qualitative process evaluation to examine facilitators and barriers to implementation of the research and intervention protocols in Nyarugusu refugee camp.

***Preliminary aims of this study (not described above) included conducting a qualitative study to identify and characterize priority mental health problems and health seeking behavior. The JHSPH IRB determined these preliminary aims to not be human subjects research as defined by DHHS regulations 45 CFR 46.102 and thus did not require IRB oversight (IRB #5706, FWA #00000287, Nov. 11, 2014)***

**II. Background and Rationale:**

The proposed research directly targets two public health areas with major gaps in evidence in the field of humanitarian settings: (1) gender-based violence and; (2) mental and psychosocial health.

**Gender-based violence**: With regard to gender-based violence in refugee settings specifically, Asgary and colleagues (2013) conducted a wide and inclusive systematic review, but did not identify any studies that evaluated prevention and reduction of GBV in this population. We therefore discuss evidence from non-humanitarian contexts and non-refugee populations, focusing on intimate partner violence as a subset within the broad category of gender-based violence. Systematic reviews have focused on two types of interventions to reduce intimate partner violence in health-care settings: (1) universal screening and (2) empowerment (advocacy)-focused counseling.

***Universal screening*** has been a contested practice (Jewkes 2013). Recent systematic reviews have found that although five screeners have shown diagnostic accuracy to detect IPV (Nelson et al. 2012), universal screening itself does not seem to be associated with a reduction in IPV rates or improved health outcomes (5 trials of low-moderate quality) (Taft et al. 2013; World Health Organization 2013b). Since effective interventions exist to target intimate partner violence, the US Preventive Services Task Force recommends universal screening of women of reproductive age and linking them with services (Moyer 2013), whereas the WHO (2013b) only recommends screening of women who show signs or symptoms of intimate partner violence.

With regard to ***empowerment counseling***, a WHO review (2013b) identified 15 studies that examined a heterogonous mix of components including: linking women to services, empowering women, and promoting safety behaviors. Findings showed that these interventions may reduce intimate partner violence in some women, but there are gaps in knowledge with regard to: if intimate partner violence interventions alone may improve mental health and quality of life outcomes; whether intimate partner violence interventions are effective when implemented with women outside of shelters/ safe houses; and if the positive results with women in antenatal clinics can be generalized to other settings. These findings are similar to findings from a Cochrane review on effectiveness of advocacy interventions to reduce IPV (Ramsay et al. 2009).

**Mental health:** A systematic review on mental health and psychosocial support interventions in humanitarian settings (Tol et al. 2011) concluded that there is moderate level evidence that more specialized treatments can reduce posttraumatic stress disorder (PTSD) in humanitarian settings (meta-analysis of 9 treatments, low statistical heterogeneity), but uncertainty with regard to other outcomes and approaches. None of the studies identified in this systematic review were conducted specifically with populations exposed to intimate partner violence. Later systematic reviews that specifically searched for evidence for interventions for sexual and gender-based violence in conflict-affected populations (Tol et al. 2013b) and refugee populations (Asgary et al. 2013) confirm this lack of studies.

However, a randomized controlled trial published by Bass and colleagues in 2013 has shown that group Cognitive Processing Therapy was associated with strong reductions in symptoms of common mental disorders (anxiety/ depression) and posttraumatic stress disorder (PTSD), as well as functional impairment among gender-based violence survivors (Bass et al. 2013). Furthermore, we may build on evidence of mental health interventions for trauma-affected populations more broadly. This evidence was reviewed by the PI as part of the development of WHO recommendations on how to manage stress-related conditions and disorders (acute stress, PTSD, bereavement) in non-specialized health care settings in low- and middle-income countries (Tol et al. 2013a; World Health Organization 2013a). With regard to PTSD, these guidelines recommend individual cognitive behavioral therapy with a trauma focus (CBT-T) and eye movement desensitization reprocessing (moderate level evidence), as well as group CBT-T and stress management (low level evidence).

Mental health and intimate partner violence are inter-related. Intimate partner violence is a known risk factor for a range of mental health conditions including PTSD, depression, anxiety, substance use, and suicidal behaviors (Howard et al. 2013; Rees et al. 2011). Mental health problems in turn, put women at a higher risk for further gender-based violence. Combining these interrelated risk factors for wellbeing may produce synergistic effects. For example, women with improved mental health may be in a better position to negotiate safety and their right to be protected from violence. Vice versa, empowerment and reductions in violence may contribute to improved mental health. Against this background, the overall aim of this study is to test an integrated intervention model that addresses both sides of this vicious cycle. We will build on the study conducted by Bass et al. with women residing in the DRC to develop and test an abbreviated evidence-based mental health intervention combined with empowerment in refugees from the DRC in Tanzania.

**III. Study Design**

1. Provide an overview of your study design and methods. The study design must relate to your stated aims/objectives. Details will be requested later. If your study also involves analysis of existing data, please complete Section XI, “Secondary Data Analysis of Existing Data” in the last part of this research plan. If your study ONLY involves analysis of existing data, please use the research plan template for secondary data analysis (JHSPH IRB Research Plan for Secondary Data Analysis of Existing Data/Specimens).

AIM 1: Pilot measurement tools assessing identified priority mental health conditions, functional impairment and intimate partner violence in Nyarugusu refugee camp

We have selected measurement tools that have been used in previous research on mental health and IPV and were consistent with the priority mental health and psychosocial problems affecting female survivors of IPV in Nyarugusu identified in our previous qualitative research (please see section V.B.4 and attached measures). However, because these tools have not been validated in Congolese refugees residing in Nyarugusu refugee camp, we plan to conduct a validation study prior to starting the randomized controlled trial. For the validation study we will recruit 60 women from zone 1 meeting the eligibility criteria for the trial to complete the screening and baseline assessment and participate in the intervention during the pilot period. The measurement validation and pilot intervention activities will be implemented by the research staff and intervention facilitators hired to complete the randomized controlled trial. For purposes of measurement validation, we will not conduct the post-intervention assessment with the pilot participants because there are no new measures introduced during these assessments. Thus, completing the screening and baseline assessments during the pilot phase will be sufficient for completing our objective of evaluating the validity and reliability of these measurement tools among women in Nyarugusu. Using pilot measurement validation data we will be able to assess inter-rater reliability, internal consistency, construct validity, test-retest reliability and face/content validity as follows.

1. *Inter-rater reliability* will be assessed by having all research assistants observe and document the same three screening and baseline pilot interviews and having one member of the research team role play as a participant. Inter-rater reliability will then be quantified by the intraclass correlation coefficient (ICC 3,10) for all measures included in the assessments. This data has already been completed as part of the role-plays conducted during training, but the intraclass correlation coefficient has not yet been calculated.
2. *Test-retest reliability* will be assessed by having the first 24 pilot participants complete the screening and baseline assessments twice (one week apart, administered by the same research assistant) prior to commencing the pilot intervention. The correlation of a given measure between these two assessments will be calculated to determine test-retest reliability. This will be evaluated once all pilot assessments have been completed.
3. *Internal consistency* will be assessed by calculating Cronbach’s alpha for each measure as well as evaluating whether the reliability substantially improves upon removal of any single item. We will also calculate item-rest correlation to assess the correlation between a single item and the total scale score (excluding the item under study). Internal consistency will be evaluated once all pilot assessments have been completed.
4. *Construct validity* will be assessed by evaluating both internal convergent and discriminant validity. To evaluate construct validity we will compare the correlation of scores on the Abuse Assessment Screen, the Demographic and Health Survey Domestic Violence Module, the Hopkins Symptom Checklist, the Harvard Trauma Questionnaire and other measures included in the screening and baseline assessment. We expect measures assessing the same construct (e.g. IPV), but on different measures will be more highly correlated (convergent validity) than that of measures assessing different constructs (discriminant validity). Using the correlations between these constructs and measures we will develop a multi-trait, multi-method matrix that will allow us to compare the relative magnitude of reliability, convergent validity and discriminant validity correlation coefficients. We hypothesize that the reliability coefficients will be highest followed by convergent validity, which will be larger than the discriminant validity coefficients. Construct validity will be evaluated once all pilot assessments have been completed.
5. *Face and content validity* has been assessed by the research team and healthcare workers in Nyarugusu during the training process. Face and content validity describe the extent to which items included in a measure appear relevant to the construct of interest (face validity) and whether they cover the entire domain of that construct (content validity). During the training and measurement development process, the research team thoroughly reviewed each measure and discussed what was intended by each item and how it relates to the construct of interest. Measures that were not relevant to the setting in Nyarugusu were removed or adapted as needed. Furthermore, clinicians and public health professionals with experience in mental health also reviewed the measures to evaluate face and content validity. Upon completion of the pilot assessment, the measures will again be reviewed by the research team and public mental health experts to evaluate face and content validity.

AIM 2: Develop and pilot implementation of the proposed integrated intervention combining and adapting elements of cognitive processing therapy (CPT) and empowerment counseling.

The 60 women that complete the pilot screening and baseline assessment will then enter into a pilot intervention cohort. The pilot intervention will resemble the intervention procedures (described in section V.C.2 Intervention Study Visits). The intervention will begin with an individual empowerment counseling session with one of the intervention facilitators followed by seven sessions (1 per week) of group integrated cognitive processing therapy and empowerment counseling. The purpose of the pilot intervention phase is to assess implementation of the intervention, as opposed to an outcome evaluation. A psychiatrist and/or psychologist from Muhimbili University of Health and Allied Sciences (MUHAS) that has been trained in the intervention will observe intervention sessions and provide feedback to the intervention facilitators throughout the study period. Participant safety protocols (e.g. suicide risk assessments, etc.) will be in effect throughout the pilot period as they would during the randomized controlled trial (see section V.B.6.f). There will be no control group enrolled during the pilot period. The pilot period has not yet started.

AIM 3: Conduct a randomized controlled trial of the intervention, to examine if the collaboratively developed intervention is associated with reductions in prioritized mental health problems and intimate partner violence, compared to a set of currently existing standard operating procedures

We plan to conduct a cluster randomized controlled trial evaluating the efficacy of the aforementioned integrated mental health treatment and intimate partner violence prevention intervention as compared with a treatment as usual control group. In Nyarugusu Refugee Camp, the setting of this study, there are 7 zones within which refugees live. These zones are further subdivided into 52 villages. Women’s groups (described in further detail below; section IV. Participants) operate within these villages and will serve as the site of recruitment and the unit of randomization. Women entering the trial will be assessed for symptoms of mental health problems, intimate partner violence and other related constructs at baseline (week 1) and one post-intervention follow-up at week 10. All measurement and data collection is based on self-report indicators reported by the participant. The intervention is designed to last approximately 2 months (8 sessions, once per week for 8 weeks) with full participation in the study lasting 10 weeks (baseline to follow-up).

AIM 4: Conduct a qualitative process evaluation to examine facilitators and barriers to implementation of the research and intervention protocols.

We plan to conduct a qualitative process evaluation of the implementation of the Nguvu intervention and research protocols within the context of the randomized controlled trial described in Aim 3. To do so we will recruit a subset of participants (n=10), both high (n=5) and low attenders (n=5) from the intervention condition to participate in a qualitative interview with a member of the research team to discuss their experience participating in the intervention related to perceived challenges, risks, benefits, operations and acceptability. We will also qualitatively interview the 10 intervention facilitators and their clinical supervisor to identify their perspectives on successes and challenges of the intervention as well as their experiences as a provider of these services. Lastly, we will conduct qualitative interviews with seven representatives from partner agencies and the community advisory board to examine structural challenges to implementation as well as the perceived impact that implementing Nguvu had at the community and organizational level. We estimate that each of these interviews will take approximately one hour to complete and will be delivered by our trained research assistant team.

1. Provide a sample size and a justification as to how you arrived at that number. If you use screening procedures to arrive at a final sample a table may be helpful.

Pilot Study:

We have chosen to include 60 women in the measurement validation and pilot intervention phase because this sample size will allow for each pair of facilitators (5 pairs in total) to run through the entire intervention starting with 12 women each. Sixty participants is also a sufficient sample size to meet the objectives of our measurement validation activities.

Randomized Controlled Trial:

Our primary outcomes are both assessed on a continuous scale, thus we conducted a sample size calculation for the randomized controlled trial intended for two-sample mean comparisons using one of our primary outcomes of interest, psychological distress. We gathered information from previous trials measuring our primary outcome(s) of interest in similar populations to estimate the following parameters used for the sample size calculation:

$$\sigma_{time1}^{2}=0.25$$

$$\sigma_{time2}^{2}=0.36$$

$$\rho=0.333$$

$$\sigma_{change}^{2}=0.55007$$

$$m=10$$

$$\mu_{1}=1.3$$

$$\mu_{2}=0.7$$

$$N=\frac{2\left( z_{\frac{\alpha}{2}}+z_{\beta} \right)^{2}(\sigma^{2})(1+\left( m-1 \right)\rho)}{{(\mu_{1}-\mu_{2})}^{2}}=\frac{2\left( 1.96+0.842 \right)^{2}(0.55007)(1+\left( 18.269-1 \right)0.333)}{{(1.3-0.7)}^{2}}=161.97$$

In order to have 80% power to detect a treatment effect of similar magnitude as previous trials that are roughly comparable in terms of intervention and population at the 0.05 significance level, we need 162 persons per group. To account for 10% attrition, we will recruit a target sample of 180 participants per group (360 total).

Process Evaluation:

We will conduct a total of 28 qualitative interviews as part of the process evaluation. We will recruit a subset of the participants that were enrolled in the randomized controlled trial (n=10) to participate in the process evaluation. We will recruit 10 intervention facilitators, their clinical supervisor (n=1) and 7 members of local partner organization to interview regarding implementation facilitators and challenges. These new participants will not be interviewed regarding personal health information and the focus of the interviews will be on program implementation.

Total Sample Size:

The total number of participants that will be enrolled in the pilot study and randomized controlled trial is 420 (60 in pilot, 360 in trial). We will recruit an additional 18 personnel who have not participated as a client in Nguvu to qualitatively interview regarding the implementation of the research and intervention protocols.

**IV. Participants**

Describe the study participants and the population from which they will be drawn. Specify the inclusion and exclusion criteria. If you plan to include children, note their ages and whether you will include children in foster care. Note if the participants are particularly vulnerable in terms of cognitive limitations, education, legal migration status, incarceration, poverty, or some combination of factors.

Congolese refugee women residing in Nyarugusu camp in northwest Tanzania reporting a past-year history of intimate partner violence and experiencing moderate to severe psychological distress will be included in this study. We will initially invite women to participate that are members of women’s groups that operate throughout the camp. Women’s groups in Nyarugusu have been organized as a mechanism to strengthen social support among women in the camp. The types of women’s groups include cooking, weaving, tailoring and microfinance. Women’s groups are formed by IRC and other agencies in the camp; however, women from the community may propose to start a women’s group to these agencies as well. Each women’s group contains approximately 11-20 women and they each elect a leader that organizes group meetings and activities throughout the camp. Members organize most women’s group activities, however IRC and other agencies do occasionally provide skills trainings to women’s groups at the women empowerment center or other venues throughout the camp. In addition to recruitment through women’s groups, we will also include women that are referred to the program so long as they meet the following eligibility criteria:

1. Inclusion criteria:
   1. 18+ years of age
   2. Female
   3. Refugee from the DRC residing in Nyarugusu refugee camp
   4. Past-year history of intimate partner violence (survivor)
   5. Experiencing moderate to severe levels of psychological distress during the past month as measured by the 25-item Hopkins Symptom Checklist and the Harvard Trauma Questionnaire (Average Score >=1.75 on either checklist, indicating close to a moderate amount of distress)
2. Exclusion criteria:
   1. Serious mental illness including intellectual disabilities that would preclude comprehension of intervention material, psychotic symptoms (e.g. hallucinations, delusions), manic symptoms and severe substance use problems (i.e. intoxication at the time of screening). Note: Participants meeting this exclusion criterion will be referred to the mental health clinic at the Tanzania Red Cross Hospital or to an IRC counselor/case manager for further assessment and referral.
   2. Imminent risk of suicide. Note: Participants meeting this exclusion criterion will be referred to one of the eight IRC counselors we have trained in suicide risk management. Research assistants are instructed to not leave participants meeting this exclusion criterion alone, if possible, until they are in the presence of the on-call IRC counselors trained in suicide risk management.

*Process Evaluation:* Ten of the participants in the process evaluation will be a random sample of high- (n=5) and low-attenders (n=5) from the Nguvu treatment condition and will thus meet the eligibility criteria described above. The eligibility criteria for the remaining 18 participants are as follows:

1. N=10: Nguvu facilitators that were trained by study staff to administer the Nguvu intervention as part of the randomized controlled trial.
2. N=1: Clinical supervisor to the Nguvu facilitators during the randomized controlled trial period
3. N=1: Field manager of the Nguvu research team
4. N=1: Focal point for protection and/or health at the UNHCR (partner agency) Kasulu field office
5. N=2: IRC (partner agency) gender-based violence program focal points in Nyarugusu camp
6. N=1: IRC (partner agency) focal points in Kasulu office
7. N=2: Community advisory board members in Nyarugusu

**NOTE**: If you are recruiting participants or receiving, accessing, or using data from a U.S. health care provider, HIPAA review is likely to be required. If you plan to bring identifiable health information from a foreign country to a U.S. covered entity (e.g., lab at the Hopkins SOM), HIPAA may be triggered. Check “yes” to the HIPAA question in the PHIRST application.

**V. Study Procedures**

In this section, provide details of your procedures, particularly as they relate to human subjects. If this is a multi-center study, make the role of JHSPH clear. If the JHSPH will serve as **data coordinating center**, indicate in the sections below which procedures JHSPH will not be performing. Additional information regarding data coordinating centers is requested in a later section. If your study will develop in phases, address each item below by phase.

A. Recruitment Process:

1. Describe how you will identify, approach, and inform potential participants about your study. Include details about who will perform these activities and what their qualifications are.

Ten research assistants have received 12 days of training on topics related to research methods, recruitment procedures, structured interviewing skills and other aspects of data collection. All research assistants have previous experience working in gender-based violence projects in the camp and/or data collection activities. Furthermore, all research assistants are Congolese refugees themselves. The leader of the research assistant team will request permission from the leaders of women’s groups in Nyarugusu to attend their next meeting. If permitted, two or more research assistants will attend the women’s group meeting and read a structured recruitment script that provides basic information about the study. The recruitment script does not explicitly state that the intervention is targeting women with a history of intimate partner violence and psychological distress in order to protect the privacy of individuals that express interest in participating. The research assistants request that interested individuals approach them after the women’s group meeting at which time they will provide more information about the program, including the sensitive aspects of it, and conduct the screening interview for eligibility.

As mentioned previously, we will also enroll women referred to the study if they meet all eligibility criteria. During our formative research we have learned that it is very common for information about programs offered throughout the camp to be spread by word-of-mouth. We would like to allow women in Nyarugusu that are eligible for the study, but not part of a women’s group to be able to participate. Thus, if a woman does learn about the study and elects to invite a woman that she knows and think could benefit to an assessment appointment, we will not exclude her. Women that engage with the study in this manner will undergo the same consent, screening, assessment and intervention procedures as women recruited through women’s groups. However, we do anticipate recruiting the majority of our participants from women’s groups as these are our primary recruitment source. To protect the privacy and safety of women in Nyarugusu, we will not be advertising or providing study contact information to anyone besides the study participants and staff/investigators.

*Process Evaluation:* Participants in the process evaluation will be approached by members of the research team and asked if they would be willing to conduct an interview regarding the Nguvu program with a focus on experiences with and recommendations for implementation. The 10 participants from the Nguvu trial will be randomly selected using a blocked design based on Nguvu intervention attendance. The remaining participants will be identified by study staff based on their roles in the Nguvu program or a particular partner agency. All participants must provide oral consent prior to proceeding with the interview.

2. Address any privacy issues associated with recruitment. If recruitment itself may put potential participants at risk (if study topic is sensitive, or study population may be stigmatized), explain how you will minimize these risks.

The recruitment script does not include any information about the target population beyond the fact that we are interested in including women for a women’s health intervention. We do not share that this intervention targets women with a history of intimate partner violence experiencing psychological distress until the individual screening process is initiated. The screening and informed consent, which is the time when more detailed information about the intervention is provided to women, is done 1-on-1 in a private setting so as to protect the privacy of the potential participant. We also chose to recruit from women’s groups in Nyarugusu because we believe these will be safe spaces where only women are present. Furthermore, these women’s groups were developed in part to strengthen women’s social networks and support.

B. Consent Process:

1. Describe the following details about obtaining informed consent from study participants. If a screening process precedes study enrollment, also describe the consent for screening.

- Who will obtain informed consent, and their qualifications
- How, where, and when the consent discussion(s) will occur
- The process you will use to determine whether a potential participant meets eligibility criteria
- Whether you will obtain a signature from the participant or will use an oral consent process
- Whether you will obtain a legally authorized representative’s signature for adults lacking capacity
- If children are included in the study, if and how you will obtain assent from them
- If children are included in the study, how you will obtain permission for them to participate from their parent, legal guardian, or other legal authority (if child is in foster care or under government supervision.)
- If you are seeking a waiver of informed consent or assent, the justification for this request
- Whether you will include a witness to the consent process and why
- If the language is unwritten, explain how you will communicate accurate information to potential participants and whether you will use props or audio materials.

*Consent for Screening (Oral)*

Prior to screening, one of the research assistants will obtain oral informed consent from the potential participant. During the research training all participants received 1.5 days of training in informed consent and related issues surrounding research ethics. All members of the research team are Congolese refugee women that have varying levels of experience working in gender-based violence programming and research. The consent discussion will occur in a private place either in the research team office or another private location requested by the participant. The oral consent describes the aims of the project, the purpose of screening, potential risks and benefits of screening, and protections for the patient (e.g. confidentiality, autonomy). The screening is estimated to take 20-30 minutes and is in the form of a structured interview. Based on the participant’s responses, the research assistant will be able to complete an eligibility assessment worksheet to determine whether the participant is eligible for enrollment into the trial. Adults lacking capacity to consent for the screening process will not be allowed to have a legally authorized representative provide consent at this phase. No children will be included in the study as per our eligibility criteria. There will be no witnesses included in the consent process.

*Consent for Enrollment (Written)*

Once participants are notified of their eligibility they will have the opportunity to continue with the baseline assessment or reschedule this for another time, if they are eligible for the trial. The baseline assessment is preceded by a written consent process that goes into much greater detail about the procedures for the trial (e.g. the intervention format), the time commitment and expectations, detailed risks and benefits of participation, as well as protections and contact information for investigators and IRB officials in case the participant has questions or concerns. This written consent phase will also be done 1-on-1 between a research assistant and a participant (there will be no witnesses). We will only consider consent to be obtained if the participant provides a signature indicating their voluntary participation or, in cases where the participant is not able to write, they will provide their thumbprint. No children will be included in the trial as per our eligibility criteria.

Figure 1. Enrollment Procedures

Recruitment

Oral consent for screening

Screening

Written consent for enrollment

Baseline assessment

*Process Evaluation (Oral Consent)*

All participants included in the process evaluation must provide oral consent to a member of the research team. The consent discussion will occur in a private place unless the participant requests otherwise and is comfortable discussing the themes of the process evaluation, which do not include personal or sensitive health information, in a public setting. The oral consent describes the purpose of the process evaluation, potential risks and benefits of participating, and protections for the patient (e.g. confidentiality, autonomy). The process evaluation is estimated to take 45-60 minutes and is in the form of an unstructured interview.

2. Identify the countries where the research will take place, and the languages that will be used for the consent process.

| Country | Consent Document(s)  (adult consent, parental permission, youth assent, etc.) | Languages |
| --- | --- | --- |
| Tanzania | Oral consent (screening) | Kiswahili |
| Tanzania | Written consent (enrollment) | Kiswahili |
| Tanzania | Oral consent (process evaluation) | Kiswahili |

*Note:* Congolese refugees in Tanzania speak Kiswahili, French and, in fewer cases, English. Kiswahili tends to be the language commonly spoken and it has developed into a hybrid form of Kiswahili incorporating elements of Tanzanian and Congolese Kiswahili. We have spent substantial time working with the research team and translators from Muhimbili University of Health and Allied Sciences (MUHAS) to adapt and translate our measures and consent form such that they reflect this type of Kiswahili (see certificate of translation).

C. Study Implementation:

Answer the following:

1. Describe the procedures that participants will undergo. If complex, insert a table below to help the reviewer navigate.

Recruitment and Screening: Contact with participants begins at recruitment. Participants will be recruited from women’s groups or on a referral basis at which time they will be invited to participate in a screening interview, which is preceded by an oral consent process.

Enrollment and Baseline Interview: If eligible, the research assistant then seeks written consent for enrollment followed by the baseline interview, which may directly follow screening or be rescheduled for another time in the upcoming week.

Randomization and Intervention: Given that this is a cluster-randomized trial, the randomization of women’s groups (unit of randomization) will occur prior to recruiting any participants. Thus, if a participant completes the baseline assessment, the intervention team (which works independently from the research team) will identify whether that person belongs to a women’s group that has been randomized to the intervention (vs. control) condition. If in the intervention group, a member of the intervention team will make contact with the participant and schedule their first intervention session. There will be eight sessions that take place once per week for eight weeks.

Follow-up Interview: The week following the intervention, the participant is then asked to complete a follow-up interview. If the participant belongs to a control women’s group then all procedures, with the exception of the intervention, will be the same.

Process Evaluation: A subset of participants will participate in a qualitative process evaluation. In addition to this subset, members of the research and intervention team as well as representatives from partner agencies will be recruited to provide their experience with the implementation of Nguvu.

Responsibilities of the Research and Intervention Personnel: As mentioned above (in Randomization and Intervention), the research assistants and intervention facilitators are expected to work independently to preserve blinding of research assistants to participant’s study assignment. Thus, the research and intervention teams are managed separately with Muhimbili University of Health and Allied Sciences (co-investigators Likindikoki and Mbwambo) responsible for managing the research team and the International Rescue Committee (co-investigator Bonz) responsible for managing the intervention team. Johns Hopkins (principal investigator Tol) is responsible for overseeing management across both teams and coordinating their activities. A summary of activities and which teams are responsible for implementation of these activities throughout the duration of the study is displayed below.

| **Study Activity (in chronological order)** | **JHU** | **MUHAS** | **IRC** |
| --- | --- | --- | --- |
| Randomization of women’s groups | X |  |  |
| Recruitment of participants |  | X |  |
| Screening of participants |  | X |  |
| Baseline assessment |  | X |  |
| Identifying study assignment (based on participant’s women’s group affiliation indicated in baseline assessment) |  |  | X |
| Intervention sessions 1-8 |  |  | X |
| Post-intervention assessment |  | X |  |
| Participant retention (in intervention, intervention group only) |  |  | X |
| Participant retention (assessments, intervention and control group) |  | X |  |
| Process evaluation |  | X |  |
| Analysis and dissemination | X |  |  |
| Coordination of activities | X |  |  |

JHU: Johns Hopkins, MUHAS: Muhimbili University of Health and Allied Sciences, IRC: International Rescue Committee

1. Describe the number and type of study visits and/or contacts between the study team and the participant, how long they will last, and where/how they will take place.

Research Study Visits: All participants will complete 2-4 research study visits: screening/baseline which may take place in a single or two separate study visits, a post-intervention interview and possibly, if randomly selected, a qualitative process evaluation interview. The screening is estimated to take approximately 20-30 minutes. The baseline and post-intervention interview are estimated to take approximately 1.5 hours. The qualitative process evaluation is estimated to take between 45-60 minutes. All research visits will take place either at the research team office, which is located in the International Rescue Committee education center in the camp, or at a private location of the participant’s choice. Participants randomized to the intervention will also have 8 intervention visits. Intervention visits last anywhere from 1.5-2 hours each and will take place at existing International Rescue Committee private meeting spaces in the camp.

In addition to the regular study visits, a random 10% of participants will be contacted by the research team leader to confirm that the research assessments took place as intended for quality assurance and data collection monitoring purposes. The research team leader will contact selected participants preferably by phone, or in person if they are unreachable, and ask them the following questions:

1. Did a member of the study team complete the scheduled research assessment?
2. Approximately how long did the assessment last?
3. What was the name of the research assistant that conducted the interview?

We anticipate that this quality assurance procedure will last approximately 2-3 minutes and will not be the source of unnecessary burden or any additional risk to the participant.

Intervention Study Visits: If a participant belongs to a women’s group randomly assigned to the intervention condition, a member of the intervention team will contact her to schedule the first intervention session. The intervention facilitators are former/current International Rescue Committee counselors involved in gender-based violence programming in the camp. The facilitators received thorough training in the Nguvu intervention, which consists of two advocacy/empowerment sessions and six cognitive processing therapy sessions. The first session of the Nguvu intervention is an individual session in which a single trained facilitator meets with the participant to discuss intimate partner violence, psychological distress and to develop a safety and emergency plan. This initial session is followed by seven group sessions of 10-12 participants led by two trained facilitators. Sessions 2-7 are adapted from the Cognitive Processing Therapy (CPT) Manual previously tested among survivors of sexual and gender-based violence in eastern DRC (Bass et al., 2013). The final session (8) integrates CPT principles and revisits the safety plan developed during session 1. A summary of session content is shown in Table 1.

Table 1. Summary of Nguvu Intervention

| **Session** | **Topic** | **Description** | **Homework Activities** |
| --- | --- | --- | --- |
| 1. Empowerment/ Advocacy | Advocacy and safety plan | - Information on IPV - Discussing psychological distress - Danger assessment - Safety plan and emergency plan | Safety plan |
| 2. CPT | Intro to CPT in Nguvu | - Introducing group rules and overview of Nguvu sessions - Stuck thoughts - Explanation of thoughts and feelings - Treatment goals | Notice and explore thoughts and distress related to IPV |
| 3. CPT | ABCs | - Introduction of ABCs - Exploring stuck points - Group relaxation | Daily practice of ABCs and relaxation task |
| 4. CPT | Stuck points and thinking questions | - Changing thoughts and feelings - Thinking questions - Exploring stuck thoughts | Daily practice of ABCs and exploration of stuck thoughts; relaxation task |
| 5. CPT | Learning safety and trust | - Introduction to safety and trust - Stuck thoughts related to trust | Daily practice of ABCs, thinking questions and changing thoughts |
| 6. CPT | Power, control and self-esteem | - Introduction to power/control issues related to self and others - Challenging questions for control issues - Self-esteem - Caring related to self and others | Daily practice of ABCs, thinking questions and changing thoughts; self-care |
| 7. CPT | CPT Review | - Discussing the impact of distressing events - Planning for the future | Daily practice of ABCs, thinking questions and changing thoughts; self-care; revise safety plan; relaxation exercise |
| 8. Empowerment/ Advocacy | Review of advocacy and safety plan | - Review safety plan - Advocacy - Coping and support methods | -- |

1. Describe the expected duration of the study from the perspective of the individual participant and duration overall.

The total duration of participation in the study is 10 weeks as described below:

| Week | 1 | 2 | 3 | 4 | 5 | 6 | 7 | 8 | 9 | 10 |
| --- | --- | --- | --- | --- | --- | --- | --- | --- | --- | --- |
| Intervention Participant | T0  T1 | S1 | S2 | S3 | S4 | S5 | S6 | S7 | S8 | T2 |
| Control Participant | T0  T1 |  |  |  |  |  |  |  |  | T2 |

T0: Screening, T1: Baseline; T2: Post-intervention interview; S1-S8: Intervention Session 1-8

1. Provide a brief data analysis plan and a description of variables to be derived.

The two primary outcomes of interest are: 1) psychological distress, and 2) intimate partner violence. Psychological distress is operationalized as the total score on self-reported symptom checklists (administered through an interview), the Hopkins Symptom Checklist and the Harvard Trauma Questionnaire, describing depressive, anxiety and post-traumatic stress symptomatology respectively. These measures were selected based on a preliminary qualitative consultation in the refugee camp. Intimate partner violence will be measured using the Demographic and Health Survey Domestic Violence Module that incorporates questions from the Conflict Tactics Scale and World Health Organization Violence Against Women Instrument. Our secondary outcome of interest is functional impairment, which is measured using items developed from formative, qualitative research.

The primary goal of the study is to evaluate whether levels of psychological distress, intimate partner violence and/or functional impairment differ by exposure to the experimental intervention. To assess this quantitatively we will construct multilevel mixed effects models that account for the clustered design (within women’s groups) and allow for longitudinal analysis of the changes in these outcomes (while accounting for within-person clustering). Given that this is a randomized controlled trial, we do not anticipate any pre-treatment confounding, but will evaluate the similarity between groups in terms of important covariates at baseline. We are also including measures of hypothesized mediators and moderators of intervention efficacy that will be analyzed as our secondary analyses.

1. Describe whether you are collecting or storing personal identifiers, and if yes, why you need them, and when and how you plan to dispose of them. Signatures on consent forms are considered to be identifiers.

There are two points in the program at which we collect and store personal identifiers. The first is after the screening at which time we ask for the participant’s contact information to allow for communication with the participant throughout the follow-up period, as well as a signature (or thumbprint) on the consent forms. Immediately following the interview, this information will be placed in a double-lock safe in the research team office. The in-country project coordinator will input this information into a password-protected file that connects the participants alphanumeric ID with their name and contact information. This is the only place that this information will be connected. Afterwards, the physical contact sheet will be shredded. The consent forms will also contain personal identifiers. These consent forms will also be kept in the double-lock safe in the research team office.

6. Answer the following **if they are relevant to your study design**:

a. If the study has different arms, explain the process for assigning participants (intervention/control, case/control), including the sequence and timing of the assignment.

The proposed study is cluster-randomized with women’s groups in the camp being defined as the clusters. Thus, randomization of women’s groups will occur before recruitment and data collection begin. The women’s groups in the camp will be randomized to intervention versus control by a faculty member in the Department of Mental Health at Johns Hopkins that has had no contact with the research field team (research assistants, in-country project coordinator). This independent faculty member will use a random allocation sequence generated by statistical software, which will remain with that independent JHSPH faculty member in Baltimore, not accessible to the research and intervention teams in Tanzania. All participants that are eligible and consent for enrollment will be asked to provide their address in the camp. Their study assignment will be determined by whether they reside in an intervention or control women’s group. The research assistants will be blind to study assignment. The in-country project coordinator, who does not conduct any data collection, will be responsible for coordinating communication with the intervention team to schedule sessions with enrolled participants belonging to intervention clusters. The process and timeline of conducting research study visits is the same between groups and will be managed by the research team.

- 1. If human biospecimens (blood, urine, saliva, etc.) will be collected, provide details about who will collect the specimen, the volume (ml) and frequency of collection, how the specimen will be used, stored, identified, and disposed of when the study is over. If specimens will be collected for use in future research (beyond this study), complete the Biospecimen Repository section below.

N/A

- 1. If genetic/genomic analyses are planned, address whether the data will be contributed to a GWAS or other large dataset. Address returning unanticipated incidental genetic findings to study participants.

N/A

- 1. If clinical or laboratory work will be performed at JHU/JHH, provide the JH Biosafety Registration Number.

N/A

- 1. If you will perform investigational or standard diagnostic laboratory tests using human samples or data, clarify whether the tests are validated and/or the lab is certified (for example is CLIA certified in the U.S.). Explain the failure rate and under what circumstances you will repeat a test. For all human testing (biomedical, psychological, educational, etc.), clarify your plans for reporting test results to participants and/or to their families or clinicians. Address returning unanticipated incidental findings to study participants.

N/A

g. If your study involves medical, pharmaceutical or other therapeutic intervention, provide the following information:

- Will the study staff be blind to participant intervention status?

Research assistants (outcome assessors) will be blind to intervention status. As is commonly the case with psychological interventions (especially with a treatment as usual control group), it will not be possible to blind the intervention facilitators or the participant to intervention status.

- Will participants receive standard care or have current therapy stopped?

All participants are able to initiate or continue to receive standard care services. Standard services consist of a set of Standardized Operating Procedures (SOPs) developed by an Inter-Agency Gender-Based Violence Working Group, consisting of UN and humanitarian non-governmental agencies. These SOPs describe a package of services for gender-based violence survivors in accordance with the international Inter-Agency Standing Committee framework (Inter-Agency Standing Committee 2007) and integrates: an immediate medical response (provided by the Tanzania Red Cross Society) including post-exposure prophylaxis where necessary and a medical exam and support; generic counseling services (provided by the International Rescue Committee); legal protection measures (provided by the International Rescue Committee, UNHCR or national justice system where necessary/desirable), which may include a short stay in designated safe houses or as a last resort, resettlement outside of the refugee camp. The latest version was updated in 2016. As part of the project, a gender-based violence service mapping was conducted with key informants to list all existing informal and formal resources that IPV survivors may rely upon as protection and support measures. We will make efforts to measure the degree to which other services were utilized by participants during the follow-up period.

- Will you use a placebo or non-treatment group, and is that justifiable?

We will use a treatment as usual control group. The separate components of this intervention (Cognitive Processing Therapy, Empowerment Counseling) have been tested in women affected by gender-based violence; however, this study is the first to evaluate these components in an integrated format and also the first to do so in a refugee population. Current services are available for intimate partner violence and some, although fewer, also exist for mental health in the camp. We do not have enough information at this point in time to know whether one study condition is superior to the other, but hypothesize that our more structured and intensive intervention (with specific cognitive therapy elements) will be more effective.

We will also facilitate referrals as appropriate for both the intervention and control group. If participants are experiencing suicidal ideation, severe psychological distress/mental health problems or report serious safety threats during the assessments or intervention the research assistants/intervention facilitators are instructed to refer them to IRC counselors for further assessment, case management and referral to the Tanzania Red Cross or another appropriate agency. During the Abuse Assessment Screen (AAS), which is the measurement tool used in the screening to evaluate past-year history of IPV, participants are instructed to refer them to the appropriate services, which is also IRC counselors and case managers. Unfortunately, this constitutes the formal services available in the camp for IPV survivors and related psychological distress. Therefore we believe that providing the control group with information about how to seek care through IRC, even if they don’t meet criteria for severe mental disorder, imminent risk of suicide or a severe safety threats (which would constitute a referral from the research assistant/intervention facilitator), is most appropriate and what we are able to offer at this time. We have intentionally selected existing IRC counselors as the intervention facilitators such that if the intervention shows evidence of efficacy, they may integrate this intervention into their standard practices which would be available to all women in Nyarugusu.

- Explain when you may remove a participant from the study.

There are a few situations in which we may remove a participant from this study. This information is also presented to the participant during the written consent for enrollment discussion. If the participant expresses imminent risk of suicide or serious mental illness, we will remove them from the study because we do not think that the intervention we are testing is suitable to manage these needs. Rather a more specialized level of care may be more appropriate and we have identified referral options in the camp through the Tanzania Red Cross, the agency responsible for health services, that is best equipped to manage these cases.

- What happens to participants on study intervention when the study ends?

The intervention was designed to be relatively short (8-weeks), but comprehensive enough to teach the relevant skills for cognitive processing that can be applied beyond the intervention period. The relatively short duration of the intervention is practical and advantageous in an unstable situation, such as a refugee camp, where there is a lot of mobility and change happening regularly. The last session of the intervention is dedicated to reviewing and re-evaluating participant’s health and safety. All participants are provided with a referral list/resource map of relevant services that are available in the camp and if the intervention facilitators believe that a participant could benefit from ongoing services, they have been instructed to make direct referrals as appropriate. Participants assigned to the intervention condition will continue to participate in the research portion of the study (10 weeks in total)

- Describe the process for referring participants to care outside the study, if needed.

We plan for all referrals for health concerns to go through the Tanzania Red Cross, which has a hospital and several health clinics in the camp. In cases of serious illness, the Tanzania Red Cross often refers to other hospitals and health centers in Tanzania. If a patient is identified as needing a referral, the research assistant or intervention facilitator will contact the in-country project coordinator who is trained to contact the appropriate health provider and escort to the participant to the hospital as needed. For cases of imminent risk of suicide or other severe mental health problems, we have trained the research team to remain with the participant and call one of the 8 International Rescue Committee counselors that have agreed to be on-call for suicide and severe mental disorder risk management.

Currently the services available for survivors of IPV and individuals experiencing severe psychological distress are quite limited. The existing referral pathway and services available for survivors of IPV include an initial needs and risk assessment conducted by IRC counselors followed referral to an IRC case manager who is responsible for facilitating access to medical services, providing legal counseling, offering material support and shelter, providing psychosocial support and discussing these services with the client. If the client needs medical services they are referred to a clinician at the Tanzania Red Cross Society (TRCS) or Medecins Sans Frontieres (MSF) for an examination. Clients with protection and security concerns and referred to the police, who may then involve the Ministry of Home Affairs and the local legal counseling agencies (e.g. WLAC) if the client would like to move forward with civil proceedings. In our early qualitative research, which involved a gender mapping project, we found that many IPV survivors choose to avoid engaging with this formal referral pathway and would prefer to resolve the violence within their family unless they are experiencing severe threats to their safety. There are fewer services available for persons with psychological distress in the camp. Psychological distress among IPV survivors is generally managed within the IRC counselor and case management services. Severe mental disorders and psychological distress, including suicidality, are typically referred to the TRCS hospital or health clinics. There is currently one psychiatrist that runs a mental health clinic at the hospital, but he tends to treat severe cases of mental disorder and refers to hospitals outside the camp if more specialized care is needed.

**VI. Data Custody, Security, and Confidentiality Protections**

The sections below describe types of data sources and how they will be protected. For the type(s) of data you will have, put an “X” in the appropriate box to the left of the section that best describes how you will minimize the risk of a breach of confidentiality for your study. Note, as appropriate, how you will record/store data. These descriptions represent MINIMAL measures; you may add more stringent protections and other relevant information in B.

**Confidentiality: The *LOSS OR THEFT* of 1) original/duplicate version of physical data collection instruments (forms, tapes, etc) or 2) physical devices containing electronic data (i.e. laptop/mobile device, external flash drive(s), is a threat to subject confidentiality.  Risk of such a loss/theft is increased during movement/transport of data (in any format), such as in a vehicle or other move.  Be sure to train anyone (co-investigators, staff, students, etc.) who might be engaged in the oversight of data handling/storage about this problem.  Some typical risk-mitigation strategies would include:**

- **minimizing the physical movement of data and/or devices containing data**
- **encrypting electronic data (especially when stored on any mobile device, including flash memory tools, phones, tablets, etc, or when transferring across networks)**
- **making use of reliable courier services (FedEx, DHL, etc) when physical transport of bulk data forms is necessary**
- **minimizing the transfer of identifiable data in physical or electronic form (i.e. removing/separating/destroying identifiable data, when physical transfer of data is necessary)**

A. Data Storage

| 1. Hard Copies of Data Collection Forms. | |
| --- | --- |
|  | This activity will not involve receiving and/or accessing hard copies of data |
|  | Data collection forms RECORD NO PERSONAL IDENTIFIERS connecting study participants, and there are no codes providing a link. Data are anonymous. |
|  | Data collection forms INCLUDE IDENTIFIERS. The forms are locked in a secure cabinet or room with limited access by authorized individuals. Forms will be kept in study team’s possession during transport and will not be left unattended in a vehicle. When possible, de-identified copies will be used for coding and analysis. |
| X | Data collection forms ARE CODED with study participants’ random study ID numbers. Codes/links between study IDs and identifiers are stored securely in a separate place (locked storage cabinet or secure electronic database.) |
|  | Other: |
| 2. Electronic Data | |
| X | The data do not contain personally identifiable information |
| X | These data are stored on a secure server protected by limited access and strong password systems. Data are coded when possible. Portable electronic devices will not contain identifiable information unless encrypted. |
|  | Other: |
| 3. Other Identifiable Data Storage, Retention, and Destruction (Audiotapes, videotapes, photographs, etc.) will be retained and stored securely (locked in cabinet or room) until: | |
|  | Transcription is complete, then will be destroyed. |
| X | Analysis is complete, then will be destroyed. |
|  | Study is complete and file is closed. |
|  | Indefinitely. Provide justification for indefinite retention: |
| 4. Existing Biospecimens to be used in this study: N/A | |
|  | HAVE NO PERSONAL IDENTIFIERS. |
|  | INCLUDE IDENTIFIERS AND ARE CODED; the PI will not have access to the link or code connecting the identifiers to the specimens. |
|  | INCLUDE IDENTIFIERS, and the PI has access to those identifiers or to the link/code connecting specimens to individuals. The identifiers and/or code will be stored securely until the study is complete. |

B. Certificate of Confidentiality

Will the study data stored in the United States be protected by a Certificate of Confidentiality? If yes, explain who will apply for and maintain the Certificate. (<http://grants.nih.gov/grants/policy/coc/appl_extramural.htm>)

N/A

C. Data Security and Sharing

PIs have the responsibility for responsible stewardship of data and protecting data confidentiality. This responsibility includes protecting physical custody of the data, storage and sharing with appropriate data use agreements that contain the appropriate security provisions. Describe any additional plans beyond those identified in the table that you have for storing and sharing the study data and/or materials, and how responsibility for the data will be managed. Include the following details:

1. Where will the study data be stored?

2. Who controls access to the data?

3. Will data be shared only if de-identified?

4. What additional (if any) security controls will be in place?

The research team has two offices, one inside the camp and one in the nearby town of Kasulu. Data kept within the camp will be stored in a double-lock safe for a maximum of 48 hours. Within that 48 hour period the in-country project coordinator will transport data to the main office in Kasulu where the data will be stored in a locked cabinet. The only people with access to the safe are the in-country project coordinator and the one research assistant that has been elected as the lead research assistant by the research team. The in-country project coordinator is the only person with access to the data in Kasulu. The in-country project coordinator will permit access to the data assistant who is responsible for data entry and management. The data that is shared with the data assistant will be entirely de-identified and will be organized by an alphanumeric study identification code. There is a single Excel database that connects the study identification code with personal identifying information. This Excel database is controlled entirely by the team leader and no other personnel, besides study investigators if necessary, will have access to this information.

**VII. Risks of the Study**

1. Describe the risks, discomforts, and inconveniences associated with the study and its procedures, including physical, psychological, emotional, social, legal, or economic risks, and the risk of a breach of confidentiality. These risks should be described in the consent documents.

Risks related to participation in the study include discomfort discussing sensitive topics and safety of the participant. First, the research interviews as well as participation in the intervention require extensive and detailed discussion of sensitive topics including a detailed history of one’s experience with intimate partner violence, symptoms of psychological distress and other potentially traumatic events. Discussing these topics has the potential to make the participant uncomfortable or even, in some cases, result in re-experiencing traumatic events and related sequelae. However, it is our experience that most participants experience the ability to discuss materials related to (threats to) their wellbeing with well-trained research assistants as cathartic. In addition, the core of the Cognitive Processing Therapy component of the intervention is to manage these thoughts such that they can be reframed to avoid experiencing negative emotional reactions. Both the research and intervention team has been instructed on how to deal with participants that appear particularly distressed. In these cases, the in-country project coordinator will facilitate a referral to a gender-based violence protection worker or health provider whichever seems appropriate.

There are also safety risks related to participating in the study. There is the potential that someone (e.g. a husband/partner, family member) may find out that a woman is participating in the Nguvu intervention and furthermore that the Nguvu intervention is an intervention targeting women experiencing intimate partner violence in the past year. This might be aggravating to a husband/partner or other person that may have been the perpetrator of this violence or other community members that may see participating as disrespectful to the woman’s relationship. To minimize these risks we have designed the study in such a way that recruitment, interview and intervention sites are all in neutral locations that are common for women to frequent or in a private place where the woman feels comfortable – so that suspicion is not raised. Moreover, intervention facilitators and research assistants review participant’s safety during intervention sessions and research assessments respectively. We also provide both the intervention and control participants with a resource map/referral chart that includes contact information for agencies in case of emergencies as well as to receive other intimate partner violence response services – so that in effect, the study will increase safety and safety monitoring of respondents.

1. Describe the anticipated frequency and severity of the harms associated with the risks identified above; for example, if you are performing “x” test/assessment, or dispensing “y” drug, how often do you expect an “anticipated” adverse reaction to occur in a study participant, and how severe do you expect that reaction to be?

Slight discomfort discussing sensitive issues surrounding intimate partner violence and mental health may be common (e.g. 1 in 5 women), but we do not anticipate that this level of discomfort will get to a concerning level of distress for most women. In 1 out of 30 women, more serious psychological distress may be experienced that requires stopping the interview. As mentioned above, research assistants have been trained in responding to distress with empathetic reactions. With all women showing distress it will be reiterated that participants can choose not to answer any questions, take a break whenever they want, or stop the interview at any time. It is our experience that among women with whom an interview was stopped, most prefer to continue after a break and an opportunity to experience and explain their distress with research assistants. Issues surrounding safety compromised by participation are also expected to be infrequent. Women in Nyarugusu are generally actively involved in women’s groups and other activities and we do not believe that attending a meeting once per week (for the intervention group) and three research assessments will seem abnormal to family and community members.

1. Describe steps to be taken to minimize risks. Include a description of your efforts to arrange for care or referral for participants who may need it.

Participants that become highly distressed during research assessments will be referred to an International Rescue Committee (IRC) gender-based violence counselor and/or health provider at the Tanzania Red Cross hospital in the camp. In situations where suicidal ideation with a plan or serious intent is expressed, the research assistant and/or in-country project coordinator will accompany the participant to the hospital.

Our strategy to address the safety risk involves both prevention and screening. For prevention we will design the intervention and research assessment implementation in such a way that identifying the program as an intimate partner violence program is highly unlikely. We are implementing the intervention using existing International Rescue Committee staff. The International Rescue Committee is an international non-governmental organization providing community services to most, if not all, refugees in the camp. We also plan to hold intervention sessions and research assessments in spaces provided by the International Rescue Committee that are normally frequented by women for activities not necessarily related to gender-based violence (e.g. ‘empowerment’ activities, income generation activities, basic literacy training, women’s health issues, etc.). In regards to screening, the intervention facilitators will assess safety during each session. The research team also inquires about intimate partner violence and safety during their assessments. If there is reason to believe that the participant is at an elevated level of risk that requires immediate response, they will refer the participant to existing gender-based violence services, including the women’s shelters and other International Rescue Committee services in the camp according to existing SOPs.

1. Describe the research burden for participants, including time, inconvenience, out-of pocket costs, etc.

The research burden involves 3-4 assessments ranging from 30 minutes to 1.5 hours each. If the participant elects to complete the baseline assessment during the same visit as the screening, they will only have two assessments, which occur at baseline and 10 weeks after enrollment. If they prefer to conduct these interviews on separate occasions the baseline interview will take place within a week from the screening and will be followed by the post-intervention assessment. The only out-of-pocket expense relates to transportation. The camp is quite large and can take approximately one hour to travel by foot from Zone 7 (northwest) to Zone 1 (southeast). If transportation challenges would preclude participation or result in out-of-pocket expenses to a participant, the research team will provide transportation vouchers, but this is not expected to occur.

1. Describe how participant privacy will be protected during data collection if sensitive questions are included in interviews.

Besides the contact sheet completed during the screening process and the consent forms, none of the forms collected during interviews will contain the participant’s name or other contact information (besides a random alphanumeric identification code). This identification code will only be connected to the participant’s name in a single excel file that is password protected. Access to this file is restricted to the in-country project coordinator and the study investigators (if necessary). We plan to secure all data, even de-identified, in a safe in the camp or a locked cabinet in the Kasulu office.

**VIII. Direct Personal and Social Benefits**

1. Describe any potential direct benefits the study offers to participants (“payment” for participation is not a direct personal benefit).

Participating in the intervention is hypothesized to reduce psychological distress and intimate partner violence, which is a direct benefit to participants in the study. Furthermore, people in the treatment as usual condition will also receive a referral and resource list that will help them identify existing services they may benefit from in the camp. Additionally, it may also be therapeutic for the participants to discuss their experiences with the research team during the assessments in a private setting.

1. Describe potential societal benefits likely to derive from the research, including value of knowledge learned.

This is the first study to evaluate an integrated mental health and empowerment intervention for survivors of intimate partner violence in a refugee camp. If the intervention shows evidence of efficacy we have designed it such that it can be directly integrated into existing services for women in Nyarugusu refugee camp (i.e., training of nonspecialized community workers in a short intervention using a manual reflecting non-technical language). We have hired and trained staff working previously as gender-based violence counselors for the International Rescue Committee in attempt to promote sustainability and capacity in this setting. Furthermore, if efficacious this program could inform practices recommended by the UN Refugee Agency (UNHCR) and other agencies working with refugees for addressing intimate partner violence and psychological distress in refugees in other settings. However, given that this is not a wait-list controlled trial we cannot guarantee that participants in the control arm will receive the Nguvu intervention after the trial has been completed. A dissemination workshop has been planned upon completion of the trial to share results of the study with local and international partners with the purpose of discussing opportunities to improve the intervention and/or integrate it into existing practices. Sustainable implementation of the Nguvu intervention that would allow for control participants to receive the intervention will require additional funds, coordination and effort that we cannot guarantee through this research study.

**IX. Payment:**

1. Describe the form, amount, and schedule of payment to participants. Reimbursement for travel or other expenses is not “payment,” and if the study will reimburse, explain.

The participants will not receive any monetary payment for their participation in the study. The Tanzania national refugee policy specifically prohibits payment of refugees residing in Tanzania outside of small incentives. Thus we plan to provide participants with a small hygiene item (e.g. soap, toothbrush) at each of the three assessments. We will also provide a transportation voucher if distance to the intervention or assessment sites would preclude participation.

1. Include the possible total remuneration and any consequences for not completing all phases of the research.

There are no consequences for not completing all phases of the research. Since no monetary remuneration will be provided, this will not be affected by attrition or adherence to study procedures.

**X. Study Management**

A. Oversight Plan:

1. Describe how the study will be managed.

Figure 1. Study Management Organogram

The principal investigator, Wietse Tol, PhD, is responsible for overseeing all aspects of the study. The Johns Hopkins staff that directly report to Dr. Tol are the trainers (Amy Joscelyne, PhD; Marian Tankink, PhD), the research and psychosocial officers (Claire Greene, MPH; Daniel Lakin, MA), the Intervention Consultants (Debra Kaysen, PhD; Reg Nixon, PhD; Agnes Tiwari, PhD; Susan Rees, PhD) and the Research Design Consultant (Judy Bass, PhD MPH). Drs. Joscelyne and Tankink developed and adapted the intervention manual and trained the intervention facilitators. Daniel Lakin contributed to intervention manual development. Drs. Kaysen, Nixon, Tiwari and Rees provided thoughtful feedback and revisions to the manual based on their expertise and experiences in intervention development for Cognitive Processing Therapy and Empowerment Counseling. Claire Greene is responsible for assisting to manage the field research operations and trained the research team. Dr. Bass has served as a consultant on research design and will be responsible for randomization.

Co-investigator, Dr. Peter Ventevogel, MD, oversees the UN Refugee Agency (UNHCR) team. Elizabeth Morrissey is the UNHCR sexual and gender-based violence consultant and has facilitated operations and developing relationships with partners in the camp. Edna Moturi, the UNHCR Public Health Officer has similarly advised us on the health system in the camp and helped to facilitate connections with relevant partners.

Co-investigators Dr. Samuel Likindikoki, MD, and Jessie Mbwambo, MD, oversee the Muhimbili University of Health and Allied Sciences (MUHAS) team. The MUHAS team is responsible for research activities. Lusia Misinzo, the In-Country Project Coordinator, manages the research team (research assistants and data assistant) and related activities in Kasulu. Tasiana Njau, MA, is a clinical psychologist at MUHAS that will be assisting with training as well as intervention supervision.

Co-investigator Annie Bonz, MA, oversees the International Rescue Committee (IRC) team. Currently Ms. Bonz is on maternity leave and is currently being covered by Ashley Nemiro. The IRC team consists of the co-investigators (located at IRC headquarters in New York City), Girogio Faedo (Deputy Director of IRC Tanzania), Timothy Mwebe Paul (Director of IRC Field Office in Kasulu), and the intervention facilitators.

The co-investigators hold a monthly call and the individual teams have meetings/calls at least once per week related to the proposed study. Furthermore, there are also meetings across these teams. The MUHAS, IRC and UNHCR personnel meet every 2 weeks to coordinate activities. JHU, being the only entirely out of country partner, has personnel in the field at least half of the time and will have a representative in the field throughout the duration of the trial.

As shown in Figure 1, each organization (e.g. Johns Hopkins, MUHAS, IRC and UNHCR) is responsible for managing different components of the study. Johns Hopkins has been responsible for training the research and intervention field teams, developing the intervention, designing the pilot study and randomized controlled trial and coordinating all other study activities. MUHAS is the primary in-country research partner and is responsible for managing the research personnel and activities as well as intervention supervision. IRC is responsible for managing the intervention facilitators and supporting intervention activities by providing access to intervention space/offices and logistical support. UNHCR is responsible for facilitating and coordinating the relationships between Nguvu staff and other operations and agencies in Nyarugusu and ensuring that the project is aligning with the needs and priorities of the camp.

2. What are the qualifications of study personnel managing the project?

The principal investigator, Dr. Tol, holds a PhD in public mental health and is an Assistant Professor in the Department of Mental Health at Johns Hopkins Bloomberg School of Public Health. Co-investigators from UNHCR (Dr. Ventevogel) and MUHAS (Drs. Likindikoki and Mbwambo) hold MDs with specialization in psychiatry. Dr. Ventevogel is the Senior Mental Health Officer at UNHCR and technically supports all UNHCR mental health activities globally. Drs. Likindikoki and Mbwambo are practicing psychiatrists and professors at Muhimbili University of Health and Allied Sciences/Muhimbili National Hospital. The co-investigator from the IRC is a licensed mental health counselor and currently serves as an international mental health technical advisor for the International Rescue Committee.

3. How will personnel involved with the data collection and analysis be trained in human subjects research protections? (Use the JHSPH Ethics Field Training Guide on our website.)

All members of the research team received 1.5 days of research ethics training adapted from the JHSPH Ethics Field Training Guide. They were also trained by a certified trainer in FHI research ethics training program. All other members of the research team have CITI training certification.

1. If the PI will not personally be on-site throughout the data collection process, provide details about PI site visits, the supervision over consent and data collection, and the communication plan between the PI and study team.

The principal investigator has previously made three site visits with plans for additional visits during the pilot and trial phases. Dr. Tol organizes a weekly field operations call with the in-country project coordinator (Lusia Misinzo), the research officer (Claire Greene) and the MUHAS co-investigators (Drs. Likindikoki and Mbwambo).

B. Recordkeeping:

Describe how you plan to ensure that the study team follows the protocol and properly records and stores study data collection forms, IRB regulatory correspondence, and other study documentation. For assistance, contact [housecall@jhsph.edu](mailto:housecall@jhsph.edu).

The pilot phase will serve the dual purpose of testing implementation of the intervention and also research assessment procedures, including recordkeeping. The in-country project coordinator is responsible for collecting data collection forms from the double-lock safe located in the camp within 48 hours and the data assistant is then expected to enter the data within 72 hours from the time that the data arrives in Kasulu. During the pilot phase and the beginning of the trial, the in-country project coordinator will travel to the camp daily to hold end-of-day meetings with the research assistants to review the data that was collected during that day and identify any sources of confusion and/or error. The in-country project coordinator will be responsible to review all data collection forms prior to data entry (ideally at the end of each data collection day) to evaluate completeness and identify any errors as soon as possible. During the pilot phase the research officer will also enter data to compare with the data assistant and identify any inconsistencies in data entry.

C. Safety Monitoring

1. Describe how participant safety will be monitored as the study progresses, by whom, and how often. Will there be a medical monitor on site? If yes, who will serve in that role?

The Data Safety Monitoring Board (DSMB) will monitor participant safety regularly over the course of the study. As described below, the DSMB will be comprised of persons at the study site (Kasulu, Tanzania) as well as persons in Dar es Salaam, Tanzania and Baltimore, MD, USA and will communicate monthly via telephone/Skype. For urgent issues that occur between DSMB meetings, the in-country project coordinator, Lusia Misinzo, will be responsible for reporting these incidents to study investigators Dr. Tol, Dr. Mbwambo and Dr. Likindikoki. We have built in safety assessments throughout the research assessments and have also discussed the importance of evaluating safety in the intervention sessions. Thus, the research and facilitator teams will also be responsible for safety monitoring. For serious safety concerns, such as suicide, we have identified a counselor from the International Rescue Committee that has agreed to provide on-call services as needed. Unanticipated safety concerns will be reported to the in-country project coordinator and discussed immediately with co-investigators in Tanzania to determine an appropriate response.

2. If a Data Safety Monitoring Board (DSMB), or equivalent will be established, describe the following:

a. The DSMB membership, affiliation and expertise.

The DSMB will consist of 1 person from Johns Hopkins Bloomberg School of Public Health that is not affiliated with the Nguvu project, 2 persons from Muhimbili University of Health and Allied Sciences in Dar es Salaam Tanzania that are also unaffiliated with the Nguvu project and a representative from the International Rescue Committee in Kasulu, Tanzania. All DSMB members have experience and formal training in research methods and research ethics that will allow them to critically analyze all available data and determine whether participants are safe, protected and the trial should proceed.

b. The charge or charter to the DSMB.

The goal of the DSMB is to periodically review and evaluate the accumulated study data for participant safety, study conduct and progress, and, when appropriate, efficacy. The DSMB will make recommendations to the study management team concerning the continuation, modification or termination of the trial.

c. Plans for providing DSMB reports to the IRB.

The DSMB will meet monthly over Skype/telephone, but may be called together for urgent cases. During these meetings, the DSMB will review interim/cumulative data for evidence of study-related adverse events, evidence of efficacy according to pre-established statistical guidance (if appropriate), data quality, data completeness, data timeliness, adequacy of compliance with goals for recruitment and retention (including those related to the participation of women and minorities), adherence to the protocol, factors that might affect the study outcome or compromise the confidentiality of the trial data (e.g. protocol violations, unmasking), and factors external to the study that may impact participant safety or ethics of the study (e.g. scientific or therapeutic developments). A report summarizing their findings and discussion will be produced after each meeting. The DSMB will provide quarterly reports to the IRB summarizing results from the prior three meetings. This report will only be shared with the study investigators if the DSMB concludes that the trial must be halted. If the DSMB makes this determination they will share this information with the IRB immediately regardless of the reporting timeline.

3. Describe plans for interim analysis and stopping rules, if any.

Participant safety will be monitored throughout the study, both by the research team (during assessments) and intervention team (during each session of the intervention). The intervention program is specifically geared towards improving safety of women experiencing intimate partner violence. All study staff and members of partner organizations may note concerns regarding participant safety or other unanticipated problems and adverse events. Concerns can be expressed to the In-Country Program Manager, who will immediately notify an established DSMB. The DSMB will be formed by the In-Country Project Coordinator, a member of the GBV Working Group and the International Rescue Committee. The DSMB will review each case, and formulate recommendations that will be followed-up by the In-Country Project Coordinator. For medical issues, the DSMB will connect with co-investigator Likindikoki (a licensed psychiatrist) and the clinical officer of the Tanzania Red Cross Society – as per existing referral pathways already described in the camp’s GBV SOPs. The DSMB will also determine whether a case forms a serious adverse event (i.e., unanticipated risks in which there is a threat of harm to participants or others, and that are related to the study), in which case the JHSPH and local IRBs (see below) will be notified.

D**.** Reporting unanticipated problems/adverse events (AE’s) to the IRB (***all studies must complete this section***):

Describe your plan for reporting to the IRB and (if applicable) to the sponsor. Include your plan for government-mandated reporting of abuse or illegal activity.

NOTE: The IRB does not require submission for all AEs, only those that are **unanticipated, pose risk of harm to participants or others, and are related to the study**.

Only adverse events that are determined to be serious adverse events (i.e., unanticipated risks in which there is a threat of harm to participants or others, and that are related to the study) by the DSMB will be notified to the JHSPH IRB and local IRBs.

E. Other IRBs/Ethics Review Boards:

If other IRBs will review the research, provide the name and contact information for each IRB/ethics review board and its Federal Wide Assurance, if it has one (available on OHRP’s website at <http://www.hhs.gov/ohrp/assurances>).

We have received approval for this study through Muhimbili University of Health and Allied Sciences and the Tanzania National Institute for Medical Research. Please see their contact information below:

*Muhimbili University of Health and Allied Sciences IRB Office*

Professor Said Aboud, Chairman, Senate Research and Publications Committee

P.O. Box 65001, Dar es Salaam, Tanzania

Tel: +255-022-2150302/6 Ext. 1016

Fax: +255-022-2152489

Email: [drp@muhas.ac.tz](mailto:drp@muhas.ac.tz)

*National Institute of Medical Research:*

Dr. Mwelecele Malecela, Chairperson, Medical Research Coordinating Committee

Dr. Margaret E. Mhando, Chief Medical Officer, Ministry of Health, Social Welfare

3 Barack Obama Drive, P.O. Box 9653, Dar es Salaam, Tanzania

Tel: +255-022-2121400

Fax: +255-022-2121360

Email: [headquarters@nimr.or.tz](mailto:headquarters@nimr.or.tz)

F. Collaborations with non-JHSPH Institutions:

For studies that involve collaboration with non-JHSPH institutions, complete the chart below by describing the collaboration and the roles and responsibilities of each partner, including the JHSPH investigator. This information helps us determine what IRB oversight is required for each party. Complete the chart for all multi-collaborator studies.

**Insert Name of Institutions in Partner column(s); add additional columns if necessary.**

|  | JHSPH | UNHCR | MUHAS | IRC |
| --- | --- | --- | --- | --- |
| Dr. Wietse Tol | X |  |  |  |
| Dr. Peter Ventevogel |  | X |  |  |
| Drs. Samuel Likindikoki and Jessie Mbwambo |  |  | X |  |
| Ms. Annie Bonz (currently covered by Dr. Ashley Nemiro on maternity leave) |  |  |  | X |

For the following, indicate “P” for “Primary”, “S” for “Secondary” as appropriate to role and level of responsibility.) Add additional items if useful.

| 1 | Human subjects research ethics training for data collectors | X |  | X |  |
| --- | --- | --- | --- | --- | --- |
| 2 | Day to day management and supervision of data collection |  |  | X |  |
| 3 | Reporting unanticipated problems to the JHSPH IRB/Sponsor | X |  |  |  |
| 4 | Hiring/supervising people obtaining informed consent and/or collecting data |  |  | X |  |
| 5 | Execution of plan for data security/protection of participant data confidentiality, as described in Sect. 5. |  |  | X |  |
| 6 | Biospecimen processing, storage, management, access, and/or making decisions about future use (N/A) |  |  |  |  |

**COMPLETE THE FOLLOWING SECTIONS WHEN RELEVANT TO YOUR STUDY:**

**XI. Secondary Data Analysis of Existing Data – N/A**

A. Study Design

1. Describe your study design and methods. The study design must relate to your stated aims/objectives.

2. Provide an estimated sample size and an explanation for that number.

3. Provide a brief data analysis plan and a description of variables to be derived.

B. Participants

1. Describe the subjects who provided the original data and the population from which they were drawn.

2. Describe whether the data contain personal identifiers of the individuals from whom the data originated. If yes, explain why you need them, and when and how you plan to dispose of them.

3. If you are receiving, accessing, or using data from a U.S. health care provider, the need for HIPAA review is likely. If you plan to bring identifiable health information from a foreign country to a U.S. covered entity (e.g., lab at the Hopkins SOM), HIPAA may be triggered. If either of these conditions is met, check “yes” to the HIPAA question in the PHIRST application.

4. If you plan to analyze human specimens or genetic/genomic data, provide details about the source of those specimens and whether they were collected using an informed consent document. If yes, explain whether your proposed use is “consistent with” the scope of the original consent, if it potentially introduces new analyses beyond the scope of the original consent, and/or if it introduces new sensitive topics (HIV/STDs, mental health, addiction) or cultural/community issues that may be controversial.

5. Explain whether (and how) you plan to return results to the participants either individually or as a group.

C. Data Management

Describe any additional plans beyond those described in Section VI that you have for storing and sharing the study data and/or materials, and how responsibility for the data will be managed.

The use of clinical data from Johns Hopkins Hospital and its affiliates requires a security review by Johns Hopkins Medicine. If you seek to access a dataset of 500 clinical records or larger, complete the *JHM Data Security Checklist* on the IRB Website.

**XII. Oversight plan for student-initiated studies: - N/A**

A. For student-initiated studies, explain how the PI will monitor the student’s adherence to the IRB-approved research plan, such as communication frequency and form, training, reporting requirements, and anticipated time frame for the research. Describe who will have direct oversight of the student for international studies if the PI will not personally be located at the study site, with that person’s qualifications.

B. What is the data custody plan for student-initiated research? *(Note: Students may not take identifiable information with them when they leave the institution.)*

**XIII. Creation of a biospecimen repository: - N/A**

Explain the source of the biospecimens, if not described above, what kinds of specimens will be retained over time. Clarify whether the specimens will be obtained specifically for repository purposes, or will be obtained as part of the core study and then retained in a repository.

- 1. Describe where the biospecimens will be stored and who will be responsible for them.
  2. Describe how long the biospecimens will be stored, and what will happen at the end of that period.
  3. Explain whether the biospecimens will be shared with other investigators, inside and outside of JHU, how the decision to share will be made, and by whom. Include the policy on commercial use and secondary distribution. Also explain how downstream use of the specimen will be managed, and what will happen to left-over specimens.
  4. Describe whether future research using the biospecimens will include specimen derivation and processing (cell lines, DNA/RNA, etc.), genomic analyses, or any other work which could increase risk to participants. Explain what additional protections will be provided to participants.
  5. If future research could yield unanticipated incidental findings (e.g., an unexpected finding with potential health importance that is not one of the aims of the study) for a participant, do you intend to disclose those findings to the study participant? Please explain your position.
  6. Explain whether the specimens will be identifiable, and if so, how they will be coded, who will have access to the code, and whether the biospecimens will be shared in linked (identifiable) form.
  7. Explain whether the repository will have Certificate of Confidentiality protections.
  8. Explain whether a participant will be able to withdraw consent to use a biospecimen, and how the repository will handle a consent withdrawal request.
  9. Describe data and/or specimen use agreements that will be required of users. Provide a copy of any usage agreement that you plan to execute with investigators who obtain biospecimens from you.

**XIV. Data Coordinating Center: - N/A**

Complete if JHSPH serves as the Data Coordinating Center.

1. How will the study procedures be developed?
2. How will the study documents that require IRB approval at each local site be developed? Will there be some sort of steering or equivalent committee that will provide central review and approval of study documents, or will template consent forms, recruitment materials, data collection forms, etc. be developed by and provided to the local sites by the coordinating center without external review?
3. Will each local clinical site have its own IRB with an FWA? State whether the coordinating center will collect IRB approvals and renewals from the clinical centers or not; if not, explain why not.
4. How will the coordinating center provide each local site with the most recent version of the protocol and other study documents? What will be the process for requesting that these updates be approved by local clinical center IRBs?
5. What is the plan for collecting data, managing the data, and protecting the data at the coordinating center?
6. What is the process for reporting and evaluating protocol events and deviations from the local sites? Who has overall responsibility for overseeing subject safety: the investigators at the recruitment site, Coordinating Center, the Steering Committee, or a data and safety monitoring board (DSMB)? Is there a DSMB that will evaluate these reports and provide summaries of safety information to all the reviewing IRBs, including the coordinating center IRB? Please note that if there is a DSMB for the overall study, then the coordinating center PI does not have to report to the coordinating center IRB each individual adverse event/problem event that is submitted by the local site PIs.
7. Who is responsible for compliance with the study protocol and procedures and how will the compliance of the local sites be monitored and reviewed? How will issues with compliance be remedied?

**XV. Drug Products, Vitamins, Food and Dietary Supplements – N/A**

Complete this section if your study involves a drug, botanical, food, dietary supplement or other product that will be applied, inhaled, ingested or otherwise absorbed by the study participants. If you will be administering drugs, please upload the product information.

A. List the name(s) of the study product(s), and the manufacturer/source of each product.

| **Name of study product** | **Manufacturer/Source** |
| --- | --- |
|  |  |
|  |  |
|  |  |

B. List each study product by name and indicate its approved/not approved status.

| **Approved by the FDA and Commercially Available** | **Approved by Another Gov’t Entity (provide name)** | **Cleared for Use at Local Study Site** |
| --- | --- | --- |
|  |  |  |
|  |  |  |
|  |  |  |

C. If your study product has an Investigational New Drug (IND) application through the U.S. Food and Drug Administration, provide the IND number and attach the Investigators Brochure and the Drug Data Sheet available on the IRB website.

D. If your study product is a marketed drug, provide the package inserts or other product information. If the study product WILL NOT be used for its approved indication, dose, population, and route of administration, provide a detailed rationale justifying the off label use of the study product.

E. If the study product is not an FDA approved drug, and is being used without an IND (e.g., dietary supplements, botanicals, etc.), provide safety information (as applicable) and a certificate of analysis.

F. Explain who will be responsible for drug management and supply, labeling, dispensing, documentation and recordkeeping,

G. What drug monitoring and/or regulatory oversight will be provided as part of the study?

**XVI. Investigational Medical Devices – N/A**

Complete this section if your study will involve an investigational medical device (diagnostic, non-significant risk, significant risk).

A. List the name(s) of the study product(s), the manufacturer/source of each product, and whether or not it is powered (electric, battery). Provide product information. If it is electric, upload documentation of clinical engineering approval.

| **Name of study product** | **Manufacturer/Source** | **Powered?** |
| --- | --- | --- |
|  |  |  |
|  |  |  |
|  |  |  |

B. List each study product by name and indicate it’s approved/not approved status.

| **Approved by the FDA and Commercially Available** | **Approved by Another Gov’t Entity (provide name and approval information)** | **Not Approved** |
| --- | --- | --- |
|  |  |  |
|  |  |  |
|  |  |  |

C. If the investigational device is a Significant Risk Device, provide the IDE number given by the FDA, or if not under FDA jurisdiction, explain why it is appropriate to use this device in this study.

D. If you believe the investigational device is not IDE exempt under 21CFR 812.2(c), but is a “Non-Significant Risk” device considered to have an approved IDE application, provide information from the manufacturer supporting that position.

E. If your investigational device is Exempt from the FDA IDE regulations, explain which section of the code applies to your device and why it meets the criteria provided. If it is a diagnostic device, provide pre-clinical information about the sensitivity and specificity of the test and the anticipated failure rate. If you plan to provide the results to participants or their physicians, justify doing so.

**References**

Asgary, R., Emery, E., & Wong, M. (2013). Systematic review of prevention and management strategies for the consequences of gender-based violence in refugee settings. *International health, 5*(2), 85-91, doi:10.1093/inthealth/iht009.

Bass, J. K., Annan, J., McIvor Murray, S., Kaysen, D., Griffiths, S., Cetinoglu, T., et al. (2013). Controlled trial of psychotherapy for Congolese survivors of sexual violence. *New England Journal of Medicine, 368*(23), 2182-2191, doi:10.1056/NEJMoa1211853.

Howard, L. M., Oram, S., Galley, H., Trevillion, K., & Feder, G. (2013). Domestic violence and perinatal mental disorders: a systematic review and meta-analysis. [Research Support, Non-U.S. Gov't]. *PLoS medicine, 10*(5), e1001452, doi:10.1371/journal.pmed.1001452.

Inter-Agency Standing Committee (2007). IASC Guidelines on Mental Health and Psychosocial Support in Emergency Settings. Geneva: IASC.

Jewkes, R. (2013). Intimate partner violence: the end of routine screening. [Comment]. *Lancet, 382*(9888), 190-191, doi:10.1016/S0140-6736(13)60584-X.

Moyer, V. A. (2013). Screening for intimate partner violence and abuse of elderly and vulnerable adults: U.S. preventive services task force recommendation statement. [Practice Guideline

Research Support, U.S. Gov't, P.H.S.]. *Annals of internal medicine, 158*(6), 478-486, doi:10.7326/0003-4819-158-6-201303190-00588.

Nelson, H. D., Bougatsos, C., & Blazina, I. (2012). Screening women for intimate partner violence: a systematic review to update the U.S. Preventive Services Task Force recommendation. [Research Support, U.S. Gov't, P.H.S.

Review]. *Annals of internal medicine, 156*(11), 796-808, W-279, W-280, W-281, W-282, doi:10.7326/0003-4819-156-11-201206050-00447.

Ramsay, J., Carter, Y., Davidson, L., Dunne, D., Eldridge, S., Feder, G., et al. (2009). Advocacy interventions to reduce or eliminate violence and promote the physical and psychosocial well-being of women who experience intimate partner abuse. [Meta-Analysis

Review]. *The Cochrane database of systematic reviews*(3), CD005043, doi:10.1002/14651858.CD005043.pub2.

Rees, S., Silove, D., Chey, T., Ivancic, L., Steel, Z., Creamer, M., et al. (2011). Lifetime prevalence of gender-based violence in women and the relationship with mental disorders and psychosocial function. [Research Support, Non-U.S. Gov't]. *Journal of the American Medical Association, 306*(5), 513-521, doi:10.1001/jama.2011.1098.

Taft, A., O'Doherty, L., Hegarty, K., Ramsay, J., Davidson, L., & Feder, G. (2013). Screening women for intimate partner violence in healthcare settings. [Meta-Analysis

Research Support, Non-U.S. Gov't

Review]. *The Cochrane database of systematic reviews, 4*, CD007007, doi:10.1002/14651858.CD007007.pub2.

Tol, W. A., Barbui, C., Galappatti, A., Silove, D., Betancourt, T. S., Souza, R., et al. (2011). Mental health and psychosocial support in humanitarian settings: linking practice and research. *Lancet, 378*(9802), 1581-1591.

Tol, W. A., Barbui, C., & van Ommeren, M. (2013a). Management of acute stress, PTSD, and bereavement: WHO recommendations. *JAMA, 310*(5), 477-478, doi:10.1001/jama.2013.166723.

Tol, W. A., Stavrou, V., Greene, M. C., Mergenthaler, C., van Ommeren, M., & Garcia Moreno, C. (2013b). Sexual and gender-based violence in areas of armed conflict: a systematic review of mental health and psychosocial support interventions. *Conflict and health, 7*(1), 16, doi:10.1186/1752-1505-7-16.

World Health Organization (2013a). *Guidelines for the Management of Conditions specifically related to Stress*. Geneva: WHO.

World Health Organization (2013b). *Responding to Intimate Partner Violence and Sexual Violence against Women: WHO Clinical and Policy Guidelines*. Geneva, Switzerland: World Health Organization.
