# Supplementary material for: Evaluation of an integrated intervention to reduce psychological distress and intimate partner violence in refugees: Results from the Nguvu cluster randomized feasibility trial
Source: PLoS One. 2021 Jun 18;16(6):e0252982. doi: 10.1371/journal.pone.0252982 (PMC8213126; doi:10.1371/journal.pone.0252982)
Supplement: Attachment — Submitted filename: IPV and mental health feasibility trial_refugees.docx [file pone.0252982.s005.docx]

This is a paper of a feasibility trial of a mental health and IPV intervention with refugees in Tanzania. The authors were responsive to comments of the previous reviewer. The authors did an excellent job describing the results of the trial. It would be helpful to add more information to the introduction to frame the significance and innovation of this intervention for this particular population and the theoretical framework that motivated the intervention. The paper would also benefit from adding some information to the methods section on the description of the intervention itself, so that the statements in the discussion are more easily interpretable. The discussion would benefit from describing what the researchers learned about conducting IPV/mental health interventions with refugees and what recommendations they have for future intervention research with refugees.

INTRODUCTION

Intro needs to discuss background on what are the specific mental health issues and IPV needs among refugees. How do refugees’ rates of mental health and IPV compare to the general population in Tanzania or DRC?

Have there been other interventions that have been tried with the same population? What did they find? How does this intervention compare with those?

p. 5, lines 82-85: This sentence could benefit from more clarity. Who is pathologizing women? Why do we need a critical feminist analysis? Any citations for this analysis and how it should be applied here?: “Studies that indicate poor mental health in the survivor may lead to increased risk for IPV require a critical feminist and human rights analysis to avoid pathologizing and blaming women for the crime of male-perpetrated violence in relationships.”

p. 5, line 88: women with mental disorders related to IPV – what are these specific disorders?

p. 6, lines 96-97: Need rationale for why this intervention is especially necessary in low-resource, refugee settings.

The third paragraph in the Intro (page 6) is about the formative research that lead to the Nguvu intervention. This feels like it should go in the Methods.

P. 6-7, lines 111-117: this is one very long sentence that is hard to follow. It should be broken up for readability.

p. 7: lines 126-130 This info is unclear and needs better explanation/elaboration for the reader to understand the importance of the study context, the multi-sectoral nature of the intervention… .

METHODS

Suggest new paragraph at line 169: “Eligible participants were adult (18+ years) female Congolese refugees living in…”

lines 187-189, this sentence is long and could use some editing to make it more succinct: “Women recruited from women’s groups randomized to Nguvu were enrolled in the intervention, whereas none of the women who were recruited from women’s groups randomized to the control received the intervention.

Was there a minimum threshold of number of members per group, in order to have the group included in the study? (for example, if only 2 members of a group participated in the study, did you retain the group in the study?)

Were all members of a group allocated to the same condition?

It would be useful to provide a short summary of the intervention in the Methods section. The authors provide citations for the intervention description, but given that this is a paper on the outcomes of the intervention, it would be useful to know some key details about the intervention here. For example, were the sessions delivered in person? Were the sessions designed to address some group-level variables, such as social cohesion? It is important to have these things in mind when evaluating the results.

Lines 225-226: What types of legal services were available to the women? What usual care services were available?

Lines 243-245: Why was IPV captured over the past two weeks and not the past month?

Lines 249-251: What is the intervention cohort study? What is the validation study? Are these the same thing?

Lines 255-257: How was acknowledging the sensitivity of the sexual violence questions supposed to improve the test-retest reliability? The connection between these two things is unclear.

Lines 269-270: Please explain what you mean by “We examined sensitivity to change in IPV… by calculating the change in primary and secondary outcomes”. What statistical analysis were you performing to examine “sensitivity”?

Lines 300-301: It sounds a bit odd to refer to documenting adverse events as an indicator of acceptability. Adverse events, on the face of it, don’t seem like the only variable for whether participants find the intervention acceptable. Perhaps framing adverse events as a safety indicator related to acceptability would help.

Lines 308-309: What types of deviations to the study protocol were recorded?

Please describe somewhere in the measures section: what is the significance of the participants’ resettlement process? What does this variable indicate?

RESULTS

Given that there were groups that were as small as 3 people, did that interfere with the way the intervention was intended? Did the analysis control for group size?

Lines 345-347: Why is the word “appeared” used in this sentence; was it higher or not? Were these means not compared statistically? If not, why?: “The proportion of women who were not married, but living with their partner, more highly educated, and literate appeared to be higher in women’s groups allocated to the Nguvu condition…”

Table 1:

- This table packs in a lot of information and it is a bit difficult to read. I suggest reducing the amount of text, if possible. For example, under the Resettlement category, I think it is not necessary to repeat “Respondent/household” for each value listed.
- Are the individual n (%) reported? There is text saying that they are listed for each category but I’m not sure which number they refer to. Can you just add a column? For example, “Education level, cluster mean %(SD), individual n(%)”

Table 2 is a nice summary of findings.

Table 3 – could you move the M(SD) and n(%) to column headings so that you don’t have to repeat them every row?

DISCUSSION

It would be helpful in the discussion to return to the very important issue of how this kind of an intervention should work in a refugee setting. Are there specific barriers or characteristics in the refugee setting that must be addressed in future interventions? What did you learn about this particular setting from this intervention?

You mention several key components of the intervention (task sharing, Cognitive Processing Therapy) that are mentioned for the first time in the discussion. It is hard to interpret these statements without reading some background on these components earlier in the paper.
