# Supplementary material for: Evaluation of an integrated intervention to reduce psychological distress and intimate partner violence in refugees: Results from the Nguvu cluster randomized feasibility trial
Source: PLoS One. 2021 Jun 18;16(6):e0252982. doi: 10.1371/journal.pone.0252982 (PMC8213126; doi:10.1371/journal.pone.0252982)
Supplement: Attachment — Submitted filename: PLOS ONE Response to reviewer comments - 2020-09-03.docx [file pone.0252982.s004.docx]

**Response to Reviewer Comments**

**Reviewer Comments**

R1.1 Congratulations to the authors for tackling a complex and difficult topic in an extremely challenging environment. It is a Herculean task but one worth doing. The study is the result of careful published developmental work and was carefully implemented in the very constraining context. This reviewer appreciated the clarity and honesty of the discussion section. I think the lessons from the study are important for others seeking to adapt the methods to their own studies. However, I think that more needs to be explained and the lessons made clearer. The authors have addressed several of the major limitations of the study in their discussion, but I have concerns that I think need addressing before the article should be published.

**Response:** Thank you for your reflections on the paper. We have noted the important suggestions you have made and detailed our responses to each of your comments below.

R1.2 The study is described as a feasibility cluster randomized trial, but it is not sufficiently well described. It is as if it is individually randomized.

**Response:** We have rearranged the ‘Procedures’ section and added additional text to clarify the cluster randomized design of the feasibility trial. Furthermore, we have revised the flow diagram as recommended in comment R1.4 to show the randomization of clusters (i.e., women’s groups) followed by the recruitment of individual women within those clusters. We hope this is now articulated more clearly in the paper.

“***We employed a cluster randomized feasibility trial design in order to build upon the existing infrastructure of local women’s groups (i.e., clusters), which are organized at the village-level by the International Rescue Committee (20). These women’s groups are aimed at strengthening social networks and providing skills training. Women’s groups focused on a variety of skills and objectives including cooking, village savings and loans, weaving, etc. Women’s groups were randomized to the Nguvu intervention versus usual care condition using a random number generator in Stata, Version 14 with 1:1 allocation.***

We obtained a list of women’s groups from the International Rescue Committee and found that 43 of the 63 listed women’s groups were operational. In these 43 women’s groups there were 647 members. We approached the leaders of the 43 women’s groups to receive their permission to deliver a brief summary of the study and invite interested members to participate. The study was presented to the women’s group members as a study of women’s health and wellbeing to avoid interested women being identified by their peers as IPV survivors. All 43 operational clusters were randomized and agreed to voluntary recruitment, screening and enrollment of their members. ***Women recruited for the study were allocated to study condition based on the randomization of women’s groups (i.e., clusters). Women recruited from women’s groups randomized to Nguvu were enrolled in the intervention, whereas none of the women who were recruited from women’s groups randomized to the control received the intervention****.*” Pgs. 9-10

R1.3 Please provide a table describing the characteristics of the clusters. (You yourselves have described the differentiation between the trainers inside those clusters).

**Response:** We have added characteristics of clusters to table 1 and described observed differences in the results section.

“At baseline participants were 33.5 (SD=9.0) years of age and had lived in Nyarugusu for 17.6 (SD=4.8) years on average. Most participants were married and living with their partner (73.6%), Wabembe ethnicity (73.6%), literate (73.7%), and Christian (18.7% Catholic, 18.7% Methodist/Free/United, 52.3% other Christian denomination). About one-third had a secondary school education or higher. Almost all participants had children (96.7%; 4.8 children, on average). The average household size was 7.3 (SD=3.3) people. Almost all participants preferred to be resettled, but approximately one-third had begun the process. Few reported completing later stages of the resettlement process (e.g., 4.9% completed a resettlement health screen, 3.3% had participated in cultural orientation sessions). ***At the cluster level, the distribution of these characteristics differed across conditions. The proportion of women who were not married, but living with their partner, more highly educated, and literate appeared to be higher in women’s groups allocated to the Nguvu condition. The mean number of children appeared higher in clusters allocated to usual care. At the individual level,*** the distribution of these demographic characteristics was similar across participants allocated to the Nguvu and usual care conditions with few exceptions. Participants in the Nguvu condition were more likely to have a secondary education, less likely to report their religious affiliation as Methodist/Free/United, and had fewer children, on average (Table 1).” Pg. 17

R1.4 The flow chart should begin with eligible clusters, followed by women. Please add to your flowchart detail of how many women were randomized into each group, following the table with cluster characteristics and then detail of any cluster dropouts as well as those individual women in each cluster.

**Response**: We have updated the flow chart as per your suggestions so the cluster randomization precedes the description of screening and allocation of individuals. In the first paragraph of the results we describe the number of clusters that were randomized. While we did not have any clusters who refused to participate or dropped out, there was one cluster that did not have any eligible women among those screened, which we describe in the first paragraph of the results section. We also added that all clusters agreed to participate and were randomized to the methods section.

“With the exception of one of the women’s groups assigned to the Nguvu condition, at least one woman from all groups was enrolled in the study (median number of women enrolled per women’s group=6; IQR=3,10).” Pg. 16

“We obtained a list of women’s groups from the International Rescue Committee and found that 43 of the 63 listed women’s groups were operational. In these 43 women’s groups there were 647 members. We approached the leaders of the 43 women’s groups to receive their permission to deliver a brief summary of the study and invite interested members to participate. The study was presented to the women’s group members as a study of women’s health and wellbeing to avoid interested women being identified by their peers as IPV survivors. ***All 43 operational clusters were randomized and agreed to voluntary recruitment, screening and enrollment of their members***.” Pg. 9

R1.5 Please include a summary of the power calculation, effect size sought, and ICC in your methods to reach n=400 outlined in your protocol prior to statistical analyses

**Response:** We have added more details about the power calculation included in the original protocol to the methods section.

“We planned to enroll 400 participants in this study, which would allow us to identify a small to moderate effect size. ***Enrolling 200 participants from the 63 original women’s groups with approximately equal allocation to each study condition was necessary to achieve at least 80% power to detect a small to moderate effect of the intervention on mental health outcomes accounting for up to 20% attrition and an intraclass correlation ranging from 0.1-0.5 within subjects and 0.1-0.3 within cluster***.” Pg. 10

R1.6 Please alter your references for the CTS. CTS 1 has been widely criticized. ORC Macro is a website for an American development aid organization that requires login or registration access. Please provide a more accessible reference or cite CTS2 if that is the adaptation.

**Response:** We have updated the references so the original survey is accessible and have clarified that we used the CTS adaptation developed for the Demographic and Health Surveys.

“Psychological, physical, and sexual IPV were measured in the baseline and follow-up assessment using an adapted version of the Conflict Tactics Scales ***that was developed for the World Health Organization multi-country study on women’s health and domestic violence against women and the Demographic and Health Surveys*** (25-27).” Pg. 12

R1.7 P.8 (under population) you add screening questions about phys/sex violence (probably better in procedures/recruitment section. P.11 you state measurement of psych/phys & sexual violence. How was psychological violence measured.

**Response:** We moved the description of screening and eligibility to the procedures section. At screening, we used the brief Abuse Assessment Screen to measure past-year physical or sexual violence, which was an inclusion criterion. Eligible, enrolled women completed a more in-depth assessment of psychological, physical, and sexual violence using the adapted version of the Conflict Tactics Scales developed for the Demographic and Health Surveys and the WHO multi-country study. We have added details about these measures to the methods section to clarify the differences in measurement at screening as compared to baseline and follow-up assessments. We have included a reference to the original measure for the baseline/follow-up assessment, which will allow readers to see the original physical, psychological, and sexual violence questions.

“***At screening, physical and sexual violence were assessed using the Abuse Assessment Screen (24).*** Psychological, physical, and sexual IPV ***were measured in the baseline and follow-up assessment*** using an adapted version of the Conflict Tactics Scales that was developed for the World Health Organization multi-country study on women’s health and domestic violence against women and the Demographic and Health Surveys (25-27). IPV frequency was calculated as the mean reported frequency of each type of IPV (physical, psychological, sexual) over the past two-weeks.” Pg. 12

R1.8 Statistical analyses. What do you mean by intent-to-treat? Do you mean inclusion of the 275 completed cases by arm?

**Response:** By intent-to-treat, we mean ‘analyzed as randomized’ whereby participants were grouped into study conditions based on randomization as opposed to what intervention they received. This was a complete case analysis as well. We have added this information to the statistical analyses section.

“To examine whether we were able to detect between-group differences we estimated the difference in means between the Nguvu and usual care condition at the post-intervention assessment. ***These intention-to-treat, complete case analyses included participants who completed the follow-up assessment and assigned study condition membership according to randomization.*** Models included random intercepts for women’s group to account for clustering.” Pg. 14

R1.9 You did very well with maintaining the sample 88%, but you did have one in ten women dropout. Even though they were (as always) the more severe, how was missingness accounted for? Did you use any imputation?

**Response:** For this feasibility trial we were not aiming to estimate the effect of the intervention. Therefore, we did not think it necessary to impute outcome data for these participants. We explored predictions of retention in treatment and attrition from the research study as important indicators of acceptability and feasibility, respectively, and have reported these results in the paper. We agree that for a definitive trial it will be important that attrition is minimized to preserve the internal validity of the study and have mentioned this in our discussion section.

“Although we had reasonable rates of follow-up in the research interviews, we found that participants in the later stages of the resettlement process were more likely to drop out because they had been relocated outside of the camp by the time of their endline interview. This non-random process may have introduced bias into our longitudinal findings because there could have been relevant differences between participants by stage of resettlement that may impact their change in mental health or IPV over time as well as their response to the Nguvu intervention. However, the degree of attrition due to resettlement was similar between groups. ***Therefore, to preserve the internal validity of a future definitive trial in displaced populations seeking resettlement or repatriation, it will be critical to account for potential study attrition in the design and analysis****.”* Pgs. 31-32

R1.10 How was missingness taken into account for composite variables?

**Response:** Most of the missing data resulted from not completing the follow-up assessment. We had very little item-level missingness and composite variables were relatively complete. In the fully adjusted models reported in Table 5, there were only 3 additional participants excluded from these models due to item-level missingness. We added a sentence to the results mentioning this alongside the research acceptability and attrition finding as well as the sample sizes for each of the models in Table 5 to indicate missingness in the outcome models.

“Research study attrition, defined as not completing the endline interview, was low (11.6%) and was not significantly different between study conditions (9.5% in Nguvu, 13.7% in usual care; *p*=0.246). The most common reason for attrition was resettlement or relocation outside of the camp (n=18 out of 36 lost to follow-up). Similarly, the only predictor of attrition was being in the later stages of the resettlement process. More specifically, having had a resettlement health screen or participating in the resettlement orientation process was associated with a 10.8-fold (95% CI: 3.7, 32.1) and 21.6-fold (95% CI: 5.3, 88.3) increase in the odds of study attrition. ***Refusal to respond to specific assessment items (i.e., item-level missingness) was uncommon.*** Only one participant withdrew from the study, which was due to concern that her neighbors would find out about her participation.” Pgs. 22-23

R1.11 Please reference your measures in the first section as well as later on

**Response:** Thank you for noting this. We have added in-text citations to accompany all references to the measures in the manuscript.

R1.12 P.6 line 98 – Please explain or give a reference for ‘qualitative free listing’ also for ‘refugee incentive worker’ for those unfamiliar with these settings

**Response:** We added a reference for the qualitative methods and definition for ‘refugee incentive worker’ in the introduction.

“To contextualize and strengthen the relevance of this intervention within a protracted refugee setting, we conducted qualitative free listing and key informant interviews with Congolese refugee incentive workers living in Nyarugusu Refugee Camp, which is located in northwestern Tanzania ***(14). Incentive workers are refugees who undertake work related to the provision of humanitarian assistance and receive fixed compensation referred to as an ‘incentive’ (15).”*** Pg. 6

R1.13 please add numbers of your ethics review boards

**Response:** We added IRB numbers for the 3 ethical review committees who approved the protocol.

“All procedures were reviewed and approved by the Johns Hopkins Bloomberg School of Public Health Institutional Review Board (***IRB0007219***), the Muhimbili University of Health and Allied Sciences Institutional Review Board ***(2014-10-27/AEC/Vol.X/56)***, and the Tanzania National Institute for Medical Research ***(NIMR/HQ/R.8a/Vol.IX/2016)***.” Pg. 11

R1.14 Please add n= at the top of tables five and table six

**Response:** We added the sample sizes for each of the models in table 5 and the overall analysis in table 6.

R1.15 Where is table 4? You may need to relabel

**Response:** Thank you for catching this error. We have renumbered the tables appropriately.

R1.16 P.20 onwards, please give number/denominator and %s

**Response:** We now report both frequencies and percentages in the text when describing study results.

R2.1 The paper presents a ‘feasibility’ seeking cluster-randomized trial (CRT). As per the convention, no sample size justification is provided. Albeit, the paper falls short on several areas which I will focus below.

**Response:** Thank you for your review and helpful comments on the paper. As per reviewer 1’s suggestion, we have added more details about the originally planned sample size calculation, but acknowledge that this study did not achieve this sample size or level of power. Please see below our detailed responses to your suggestions.

R2.2 Description of randomization is either incomplete or missing. This is very important as the randomization in CRT is much more challenging. Please describe clearly randomization scheme. A feasibility aspect of randomization needs to be discussed.

**Response:** We have added detail to our description of the randomization and allocation process in the procedures section along with a reference providing further information about different randomization approaches, including unrestricted simple randomization, for cluster randomized trials. In the discussion we note that this randomization approach was insufficient and did not result in exchangeability. Future definitive trials should consider another randomization approach (e.g., stratified randomization) to overcome the limitations described in this paper.

“Women’s groups were allocated to the Nguvu intervention versus usual care condition using a ***simple, unrestricted randomization approach with approximately equal allocation. The random number sequence was generated in Stata, Version 14***.” Pgs. 8-9

R2.3 Why CRT is chosen as compared to simple RCT also needs to be discussed. For example what consist of a cluster unit and why individual randomization to one of the arms cannot be done should be discussed.

**Response:** We have provided a rationale for cluster as opposed to individual randomization in the methods and the discussion. In this study context women’s groups served as a safe location for recruitment and screening and women preferred to participate in groups along with their peers. To avoid contamination and to leverage the existing peer support within these women’s groups, we employed a cluster randomized design for this feasibility trial. We note the limitations of this approach, particularly covariate imbalances, in our results and discussion sections.

“We employed a cluster randomized feasibility trial design in order to build upon the existing infrastructure of local women’s groups (i.e., clusters), which are organized at the village-level by the International Rescue Committee (22). These women’s groups are aimed at strengthening social networks and providing skills training. Women’s groups focused on a variety of skills and objectives including cooking, village savings and loans, weaving, etc. ***Given the strength of these networks and the potential for contamination within women’s groups, we elected to randomize women’s groups as clusters as opposed to randomizing eligible women individually.*** Women’s groups were allocated to the Nguvu intervention versus usual care condition using a simple, unrestricted randomization approach with approximately equal allocation (24). The random number sequence was generated in Stata, Version 14.“ Pg. 8-9

“First, randomizing more clusters and/or using stratified or other randomization procedures to eliminate between-group baseline imbalances is needed to preserve the internal validity of future cluster-randomized controlled trials of Nguvu (24). ***An alternative approach is to consider individual randomization. In this study context, cluster randomization was preferred as women’s groups provided a safe setting for recruitment and screening, women preferred to attend groups along with their peers, and there was a high risk for contamination within women’s groups (22)***.” Pg. 33

R2.4 Report intra-class-correlation as that is useful for a future powered trial.

**Response:** Thank you for this suggestion. We have added the ICCs for each of the outcomes to the results section.

***“The intraclass correlation coefficient across all outcomes was low: depression (ICC=0.062), functioning (ICC=0.058), sexual IPV (ICC=0.032), anxiety (ICC=0.030), post-traumatic stress (ICC=0.026), psychological IPV (ICC=0.007), and physical IPV (ICC<0.001).”*** Pg. 24

R2.5 Following the notion of feasibility trial all results of significance and p-value must be removed. Not, a pilot or feasibility trial is not efficacy establishing trials and as a result, no claim of significance can be made. Confidence intervals are fine as well as text indicating the direction of effect size.

**Response:** Thank you for this reminder to avoid overstating the findings from this feasibility trial. We have removed p-values and references to ‘significant’/’non-significant’ results from the paper and tables.

R2.6 While no sample size justification is not necessary I wonder how authors came up with n=311! How many clusters per arm is there? Not, this is pretty large sample size for a feasibility study which typically has much restricted sample size. This issue needs clarification.

**Response:** We have revised our flow chart to include the number of clusters randomized to each study condition (see Figure 1). We also added explanation of the sample size to the methods section (Pg. 10; R1.5 comment).

R2.7 It is not clear whether authors encountered any missing data and how it is handled or plans to be handled in the powered future trial

**Response:** Thank you for noting that we did not describe missing data nor how it was handled. As noted in our reply to reviewer 1’s comments (R1.9; R1.10), most of our missing data was due to research study attrition (approximately 11% of our sample) as opposed to item-level missingness. We have added the sample size for all models in Table 4, which shows that among participants who completed the follow-up interview, only 3 participants were dropped from the fully adjusted models due to item-level missingness (see R1.10). In our discussion we elaborated on study attrition as a limitation and recommendations for addressing drop out (see R1.9). In this feasibility trial we examined correlates of attrition and found that resettlement was the primary reason and predictor of attrition, which was of comparable magnitude in both groups. Given that the objective of this feasibility trial was to examine missingness as an indication of acceptability of procedures and the feasibility of a definitive trial (not to estimate intervention effects), we did not impute missing data points or employ other missing data procedures; however, we do recommend in our discussion that this be considered for a full trial.

R2.8 Also explain how the data will be made available as per PLOS-policy.

**Response:** Our local IRB protocols specify that only the research team will have access to the data. This level of restriction is necessary given the sensitive nature of the data, the vulnerability of the population (i.e., female refugees experiencing intimate partner violence who are members of local women’s groups), and the potential identifiability of participants within Nyarugusu refugee camp (n=311 enrolled women out of our sampling frame of 647 women who are part of IRC’s women’s groups). Identification of participant who have reported intimate partner violence has the potential to put women at significant risk. We have provided the email address for the Research Data Management contact at the University of Copenhagen, who will review all data requests and will require IRB approval from the individual’s home institution in order to conduct secondary analysis of these data in effort to protect the safety of research participants.

**Editorial Comments**

E.1 Please ensure that your manuscript meets PLOS ONE’s style requirements, including those for file naming. The PLOS ONE style templates can be found at <https://journals.plos.org/plosone/s/file?id=wjVg/PLOSOne_formatting_sample_main_body.pdf> and

<https://journals.plos.org/plosone/s/file?id=ba62/PLOSOne_formatting_sample_title_authors_affiliations.pdf>

**Response:** Thank you for providing these helpful references. We have modified the formatting of the paper to comply with PLOS ONE’s style requirements.

E.2 We note that you have indicated that data from this study are available upon request. PLOS only allows data to be available upon request if there are legal or ethical restrictions on sharing data publicly. For information on unacceptable data access restrictions, please see <http://journals.plos.org/plosone/s/data-availability#loc-unacceptable-data-access-restrictions>

**Response:** Our local IRB protocols specify that only the research team will have access to the data. This level of restriction is necessary given the sensitive nature of the data, the vulnerability of the population (i.e., female refugees experiencing intimate partner violence who are members of local women’s groups), and the potential identifiability of participants within Nyarugusu refugee camp (n=311 enrolled women out of our sampling frame of 647 women who are part of IRC’s women’s groups). Identification of participant who have reported intimate partner violence has the potential to put women at significant risk. We have provided the email address for the Research Data Management contact at the University of Copenhagen, who will review all data requests and will require IRB approval from the individual’s home institution in order to conduct secondary analysis of these data in effort to protect the safety of research participants.

E.3 In your revised cover letter please address the following prompts: a) If there are ethical or legal restrictions on sharing a de-identified data set, please explain them in detail (e.g., data contain potentially identifying or sensitive patient information) and who has imposed them (e.g., an ethics committee). Please also provide contact information for a data access committee, ethics committee, or other institutional body to which data requests may be sent; b) If there are not restrictions, please upload the minimal anonymized data set necessary to replicate your study findings as either Supporting Information files or to a stable, public repository and provide us with the relevant URLs, DOIs, or accession numbers. Please see <http://www.bmj.com/content/340/bmj.c181.long> for guidelines on how to de-identify and prepare clinical data for publication. For a list of acceptable repositories, please see <http://journals.plos.org/plosone/s/data-availability#loc-recommended-repositories>. We will update your Data Availability statement on your behalf to reflect the information you provide.

**Response:** Thank you for offering to update our data availability statement. We have responded to the two issues you addressed in the cover letter.

E.4 Please include captions for your Supporting Information files at the end of your manuscript, and update any in-text citations to match accordingly. Please see our Supporting Information guidelines for more information: <http://journals.plos.org/plosone/s/supporting-information>

**Response:** We have renamed our supporting information files and added captions at the end of our manuscript.
